# Oil in the Gulf of Mexico after the capping of the BP/*Deepwater Horizon* Mississippi Canyon (MC-252) well

**DOI:** 10.1007/s11356-015-4421-y

**Published:** 2015-04-16

**Authors:** Steve R. Kolian, Scott A. Porter, Paul W. Sammarco, Detlef Birkholz, Edwin W. Cake, Wilma A. Subra

**Affiliations:** EcoRigs Non-Profit Organization, 6765 Corporate Blvd., Suite 1207, Baton Rouge, LA 70809 USA; Louisiana Universities Marine Consortium (LUMCON), 8124 Hwy. 56, Chauvin, LA 70344 USA; ALS Environmental, 5424-97 Street, Edmonton, AB T6E 5C1 Canada; Gulf Environmental Associates, 2510 Ridgewood Road, Ocean Springs, MS 39564 USA; Louisiana Environmental Action Network (LEAN), and Lower Mississippi Riverkeepers, PO Box 9813, New Iberia, LA 70562 USA

**Keywords:** BP-*Deepwater Horizon*, Gulf of Mexico, Oil spill, Maturity ratios, Diagnostic ratios

## Abstract

Evidence of fresh oil from the BP*/Deepwater Horizon* Mississippi Canyon-252 (MC-252) well was found in the northern Gulf of Mexico up to 1 year and 10 months after it was capped on 15 July 2010. Offshore and coastal samples collected after capping displayed ratios of biomarkers matching those of MC-252 crude oil. Pre- and post-capping samples were compared. Little weathering had occurred, based on the abundance of low-molecular-weight (LMW) n-alkanes and polycyclic aromatic hydrocarbons (PAHs) in the post-capping samples. The occurrence of fresh oil in offshore waters and coastal areas suggest that the MC-252 well continued to leak hydrocarbons into the Gulf of Mexico at least until 22 May 2012, the end of this study period.

**Electronic supplementary material:**

The online version of this article (doi:10.1007/s11356-015-4421-y) contains supplementary material, which is available to authorized users.

## Introduction

The BP/*Deepwater Horizon* Mississippi Canyon-252 (MC-252) oil spill escaped from the Gulf of Mexico floor 1500 m below the surface and lasted 87 days, from 20 April 2010 through 15 July 2010, leaking approximately 5 million barrels (bbl) of oil and natural gas (McNutt et al. 2011a, 2012b). During our routine coastal and offshore research trips, oil slicks were observed over a year after capping (Kolian et al. 2013). These slicks were the same color and texture as the BP oil slicks that appeared in the summer of 2010 and drifted through the same offshore and coastal areas. Other researchers observed fresh MC-252 oil near the well site in 2011 and 2012 (Dittrick 2012a, b; RestoreTheGulf.gov 2012; Aeppli et al. 2013). Oil slicks were recorded by an aircraft near and inshore of the MC-252 well site from March 2011 to February 2012 (On Wings of Care.org 2014) and a satellite recorded oil slicks near the MC-252 well site in September 2012 (RestoreTheGulf.gov 2012). After several months of oil slicks on the ocean surface, we hypothesized that the oil slicks were recently discharged. Going forward, we collected samples when we encountered oil slicks during our routine research trips and examined existing samples that were collected on previous research trips during the months after the capping.

Oil slicks that are not mixed with dispersants follow a predictable course of weathering (American Petroleum Institute [API] 1999; International Tanker Owners Pollution Federation [ITOPF] 2002; Comité Européen de Normalisation [CEN] 2011). The process is rapid in severe weather conditions but may be slow in sheltered and calm areas of water (CEN 2011). The weathering processes are illustrated in the Online Resource supplemental section on Figs. S1 and S2. The Macondo leak was unusual because the crude oil lost gaseous and some soluble compounds (C_1_ to C_8_) during its 1500-m ascent to the surface (Joye et al. 2011; Ryerson et al. 2011, 2012). Detailed discussion of observations made on the subsurface portion of the MC-252 hydrocarbon plume are described elsewhere (Camilli et al. 2010; Hazen et al. 2010; Atlas and Hazen 2011; Kessler et al. 2011; Kujawinski et al. 2011; North et al. 2011; Valentine et al. 2012; Edwards et al. 2011; Reddy et al. 2012; Socolofsky et al. 2011; Weisberg et al. 2011; Spier et al. 2013).

Fresh crude oil in the marine environment weathers by progressively losing low-molecular-weight (LMW) organic compounds that evaporate, dissolve, or degrade through a number of environmental pathways (Operational Science Advisory Team [OSAT-2] 2011; Volkman et al. 1992; USEPA 1999; Passow et al. 2012). Pugliese Carratelli et al. (2011) estimated that MC-252 surface oil traveled at an average speed of 6 km day^−1^ in the wind-driven currents in the Gulf of Mexico. Neutrally buoyant tar balls eventually wash ashore or sink into the sediments, and they usually do not travel in regional currents for longer than 9 months (API 1999; ITOPF 2002; CEN 2011).

The U.S. Department of Interior Minerals Management Service [MMS] (2000) modeled the time for large light-crude oil spills to naturally disperse on the ocean surface in 6 m s^−1^ winds in the Gulf of Mexico. Large oil spills (>5000 bbl day^−1^) can display a visible oil slick on the ocean surface 5 to 30 days depending on its tendency to form emulsion (MMS 2000). Large slicks can form emulsified oil “mousse” that is more resistant to weathering causing the oil to drift in the currents for longer periods (Belore et al. 2011; Daling 2011). For a large subsurface well discharge of 7200 bbl day^−1^, with a high tendency to form an emulsion, the time before natural dispersion was 7 to 24 days (MMS 2000). Smaller discharges of oil of 5000 bbl day^−1^ were predicted to disperse in 5 to 17 days. Modeling results of batch spills of 20,000 bbl day^−1^ showed the time to disperse the surface oil slick ranged from 5 to ≥30 days depending on the oil’s tendency to emulsify (MMS 2000).

Small discharges of oil from offshore platforms, pipelines, and natural sources are common in the Gulf of Mexico (MacDonald et al. 1993, 1996; National Response Center 2015). Small discharges of ∼50 bbl day^−1^ occur twice a month, but large discharges >5000 bbl day^−1^ are rare, occurring about once every 5 years (Eschenbach et al. 2010). Small discharges of Louisiana sweet crude (≤50 bbl day^−1^) oil do not emulsify, survive longer than a day or two, or travel far on the ocean’s surface. Out of the MC-252 well, the Louisiana sweet crude, like most light crude oils in the region, would naturally disperse relatively fast and not form an emulsion (MMS 2000); however, the MC-252 oil started to form a water-in-oil emulsion during its 1500-m ascent to the surface as the plume lost its C_1_ to C_9_ compounds (Belore et al. 2011; Ryerson et al. 2011). The MC-252 oil formed a stable water-in-oil emulsion mousse in ∼48 h (Belore et al. 2011; Reddy et al. 2012; Ryerson et al. 2012).

### Diagnostic and maturity ratios

To investigate the probability that the oil in the samples was fresh, we examined the LMW compounds of pre- and post-capping samples using gas chromatography with flame ionization detector (GC-FID). To determine the source of the post-capping samples, we investigated the PAHs and petroleum biomarkers using gas chromatography-mass spectrometry (GC-MS). The depletion of LMW n-alkanes (C_11_ to C_17_) in a sample suggests that the oil is moderately weathered by evaporation or microbial degradation (Wang and Fingas 1995; Ezra et al. 2000; Stout et al. 2002, 2005; Wang and Fingas 2003a, b; Wang et al. 2006; Hansen et al. 2007; Oil Spill Identification Network of Experts [OSINE] 2007, 2011; CEN 2011; Aeppli et al. 2012; Carmichael et al. 2012; Lui et al. 2012).

Similarly, relative abundances of LMW and high-molecular-weight (HMW) polycyclic aromatic hydrocarbons (PAHs) have been used to assess weathering (Boehm et al. 1982; Sauer et al. 1993; Wang and Fingas 1995). For example, naphthalenes (two-ring PAHs) degrade faster than phenanthrenes (three-ring PAHs) and chrysenes (four-ring PAHs) (Sauer et al. 1993). Two-ring PAHs are more soluble than higher-ring compounds; thus, the absence of naphthalenes can be an indicator that weathering has occurred.

Diagnostic Ratios of biomarkers were used to determine whether the field samples and MC-252 reference samples were from a common source. Diagnostic ratios were determined for the hopane and sterane classes because these biomarkers have been shown to be resistant to weathering (Mulabagal et al. 2013; Aeppli et al. 2014).

## Materials and methods

### Samples

**Fig. 1:**
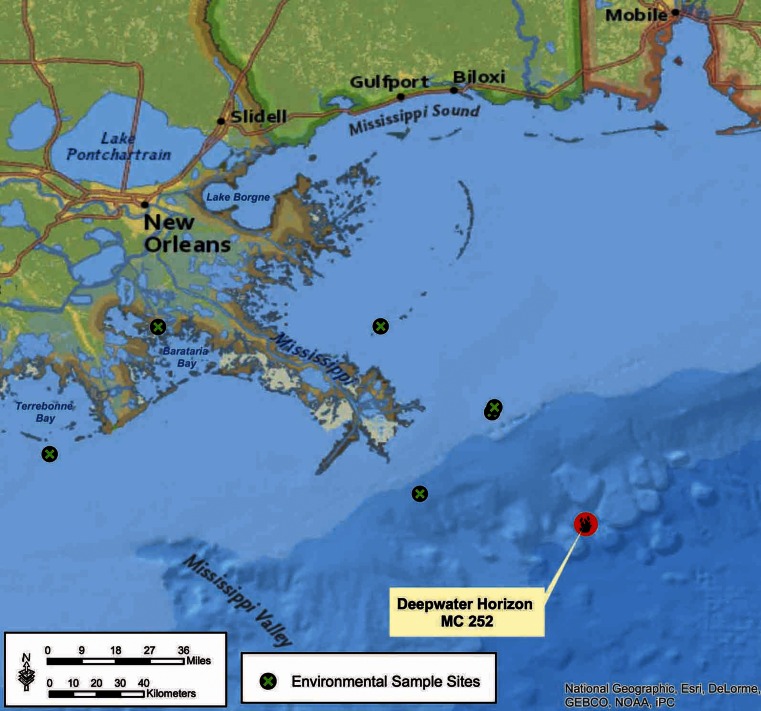
Location map of post-capping sample sites. Six samples were collected between 12 September 2010 and 22 May 2012 (59 to 677 days after capping)

The samples used in this analysis were grouped into two categories: pre-capping and post-capping environmental samples:

1. Six pre-capping samples were collected from 22 May to 10 June 2010. These consisted of oil and sediment that had washed ashore on the mainland beaches of Port Fourchon, Louisiana, and Pensacola, Florida. Data from these samples were originally included in analyses reported by Sammarco et al. (2013).
2. Six post-capping samples were collected from 12 September 2010 (59 days after capping) through 22 May 2012 (677 days after capping). Initially, we sent seven water samples stored in laboratory freezer, collected during routine research, to the lab to screen them for MC-252 oil biomarkers. We found that two of the seven contained the petroleum biomarkers hopanes and steranes, and we performed further diagnostic and maturity analysis and included them in this study. The other four samples included in this study were collected later in 2011 and 2012 when oil slicks were encountered during routine research trips. One of these samples was collected on the shores of Breton Island, LA, and the other three samples were collected from offshore oil slicks. The locations of the sample sites are presented in Fig. 1. A list containing the location, dates, sample number, and media of samples is presented in Table 1.

**Table 1:** Sample identification, collection date, location, media, and coordinates

Period	Sample ID	Date of Sample	Location	Media	Longitude	Latitude
Pre-capping	1	23-May-10	Port Fourchon, LA	Sediment	−90.1860167	29.1039667
	2	23-May-10	Port Fourchon, LA	Sediment	−90.1860167	29.1039667
	3	24-May-10	Port Fourchon, LA	Water	−90.1860167	29.1039667
	4	6-Jun-10	Gulf Coast National Seashore, FL	Sediment	−87.0054000	30.3561167
	5	7-Jun-10	West Pensacola, FL	Sediment	−87.4333500	30.2963667
	6	7-Jun-10	West Pensacola, FL	Sediment	−87.4333500	30.2963667
Post-capping	1	12-Sep-10	Venice, LA	Water	−88.7461320	29.1642100
	2	12-Sep-10	Venice, LA	Water	−88.7368940	29.1834000
	3	28-Mar-11	Breton Island, LA	Sediment	−89.1715010	29.4926670
	4	3-Apr-11	Timbalier Island, LA	Water	−90.4366640	29.0041360
	5	16-Aug-11	Denis Bayou, LA	Water	−90.0226560	29.4898940
	6	22-May-12	Venice, LA	Water	−88.7181010	30.2519380

Samples were collected in 1-L amber jars and sealed in plastic bags, cooled to <4 °C in coolers, and transferred to refrigerators or freezers for storage at temperatures of <4 °C or −20 °C, respectively, until processed. The samples collected during the pre-capping months followed the same protocol. One of the post-capping samples was collected with hydrocarbon adsorbent cloth (Dynasorb®, Dynamic Adsorbents, Inc. Norcross, GA) and was wrapped in aluminum foil, sealed in a plastic bag, and cooled to <4 °C. The sample was then transferred to a freezer and stored at −20 °C until processed. We collected photographs of the oil slicks and coordinates of the samples sites. The photographs are provided in the Field Observation section on the Online Resource supplement information link. Samples were shipped in sealed coolers overnight to the laboratory for processing along with signed chain-of-custody documents.

### Analytical methods

Sample identification and weathering analysis included two levels of analytical procedures as described in Hansen et al. (2007) and other sources (OSINE 2007, 2011; CEN 2011). Total extractable n-alkanes (C_11_ to C_60_) were measured using GC-FID (USEPA Method 3580/8000-GC-FID). PAHs, alkylated PAHs, and biomarker concentrations in water samples were measured using GC-MS USEPA method 3510/8270 (USEPA 2007), and results were provided in micrograms per liter. Sediment samples were analyzed using GC-MS USEPA method 3540/8270 (USEPA 2007), and results were provided in milligrams per kilogram.

GC-MS was used to measure concentrations of biomarker compounds in post-capping samples such as steranes and hopanes. An extract of a reference MC-252 sample was analyzed six times, and a MC-252 National Institute of Standards and Technology Standard Reference Material (NIST SRM 2779) sample was analyzed four times for comparative purposes. Biomarker diagnostic ratios have been reviewed extensively elsewhere (Peters and Moldowan 1993; Stout et al. 2002, 2005; Wang and Fingas 2003a; Peters et al. 2005a, b; Wang et al. 2005, 2006, 2007; Hansen et al. 2007; OSINE 2007; CEN 2011). The GC-FID and GC-MS analyses and diagnostic ratio analysis were performed by ALS Environmental (5424 97 Street, Edmonton, AB T6E 5C1 Canada).

#### Maturity ratios

Gas chromatogram/histogram data were used in the interpretation of weathering. Three quantitative methods were employed to analyze the data:

1. GC-FID n-alkane results were used to calculate the saturated hydrocarbon weathering ratio (SHWR). It was used to measure the relative abundance of LMW and HMW n-alkanes. The SHWR approaches 1.0 when the LMW compounds (C_11_ to C_17_) are lost to evaporation or degradation (Boehm et al. 1982).
  1 $$ \mathrm{SHWR}=\frac{\left(\mathrm{sum}\;\mathrm{o}\mathrm{f}\;\mathrm{n}-\mathrm{alkanes}\;\mathrm{f}\mathrm{rom}\;{\mathrm{C}}_{11}\;\mathrm{t}\mathrm{o}\;{\mathrm{C}}_{25}\right)}{\left(\mathrm{sum}\;\mathrm{o}\mathrm{f}\;\mathrm{n}-\mathrm{alkanes}\;\mathrm{f}\mathrm{rom}\;{\mathrm{C}}_{17}\;\mathrm{t}\mathrm{o}\;{\mathrm{C}}_{25}\right)} $$
2. The GC-MS results were used to calculate the aromatic weathering ratio (AWR), which measures the relative abundance of LMW and HMW PAHs. The AWR approaches 1.0 as LMW PAHs are lost to evaporation or degradation (Boehm et al. 1982).
  2 $$ \mathrm{A}\mathrm{W}\mathrm{R}=\frac{\left(\mathrm{total}\;\mathrm{naphthalenes}+\mathrm{flourenes}+\mathrm{phenanthrenes}+\mathrm{dibenzothiophenes}\right)}{\left(\mathrm{total}\;\mathrm{phenanthrenes}+\mathrm{dibenzothiophenes}\right)} $$
3. The GC-MS results were used to calculate the total naphthalenes-to-total PAH (TPAH) ratio. A simple indicator of early PAH weathering is the ratio of the family of naphthalenes to the total PAHs in the environmental sample (Sauer et al. 1998). The concentration of total naphthalenes was divided by the concentration of TPAHs in the environmental samples.
  3 $$ \mathrm{Naphthalenes}/\mathrm{P}\mathrm{A}\mathrm{H}\;\mathrm{ratio}=\frac{\left(\mathrm{total}\;\mathrm{naphthalenes}\right)}{\left(\mathrm{TPAH}\right)} $$

#### Diagnostic ratios and critical differences

Biomarker diagnostic ratio analyses were conducted on four of the six post-capping samples. The samples collected in 2011 and 2012 were analyzed for 11 biomarkers. In order to determine whether a given sample matched the reference sample (MC-252 oil), we calculated the absolute difference between the respective corresponding ratios. The mean ratio was also calculated for each set of ratios observed for a sample and for MC-252 reference oil. Relative differences were determined by dividing the observed means into the absolute differences. This relative difference is presented as a percentile. If the relative difference observed for a specific biomarker was less than 14 %, then the comparison was considered to be a match. If the relative difference was observed to be greater than 14 %; however, it was considered to be a non-match (CEN 2011). This approach to the analysis is more conservative than those used by Mulabagal (2013) and Aeppli (2014), who used 20 % as a limit. Diagnostic ratios used in our analysis are presented in Table S1 in the Online Resource supplemental information link. Based on the results of this multiple series of biomarker comparative analyses, we report match of the samples as “positive,” “probable,” “negative,” or “inconclusive.” Analysis was performed by ALS Environmental (5424 97 Street, Edmonton, AB T6E 5C1 Canada).

## Results

### GC-FID n-alkanes

Post-capping water samples (samples 1 and 5) displayed concentrations of LMW C_11_ n-alkanes indicating they experienced little weathering. The average first-resolved compound for all post-capping samples was C_13_. The lowest-resolved compound from the pre-capping samples was C_13_ with an average of C_14_. Post-capping sample 2 displayed a SHWR of 1.49, the highest of all of the pre- and post-capping samples, and was collected 59 days after the well was reported to be capped. n-Alkanes were not detected (ND) in post-capping sample 4, and it was excluded from the calculation of resolved n-alkanes or SHWR means. The highest pre-capping SHWR was 1.03, and the mean SHWR of the pre-capping environmental samples was 1.02 while the post-capping mean was 1.16, an increase of 13 %, indicating that as a group, they were less weathered than the pre-capping samples.

### GC-MS PAHs and alkylated PAHs

The post-capping samples displayed greater AWRs than the pre-capping environmental samples. The highest pre-capping AWR was sample 3, displaying an AWR of 1.15; the mean AWR for pre-capping samples was 1.11. The post-capping samples displayed higher AWRs, ranging from 1.03 to 2.61 (post-capping samples 6 and 2, respectively). The mean AWR for all the post-capping samples was 1.46, an increase of 29 %, indicating they were less weathered than the pre-capping samples.

### Naphthalene/TPAH ratio

The highest pre-capping naphthalene/TPAH ratio was 2.9 × 10^−2^ (sample 3) and the greatest post-capping was 4.1 × 10^−1^ (sample 2), approximately 14 times greater, indicating the post-capping sample was less weathered. All pre-capping samples contained naphthalenes or alkylated naphthalenes; however, one of the post-capping samples (sample 3) did not show concentrations of the two-ring PAH. The mean concentration of the six pre-capping samples was 1.8 × 10^−2^, and the mean concentration of the post-capping samples was 1.4 × 10^−1^, approximately seven times greater than samples collected during the spill. Table 2 presents the lowest resolved compound, SHWR, AWR, and naphthalene/TPAH ratios of each of the samples and means of the pre-and post-capping samples. Concentrations of PAHs and alkylated PAHs of pre- and post-capping samples are provided in Tables S1 and S2 on the Online Resource supplement information link.

**Table 2:** Provenance of pre- and post-capping samples

Period	Sample ID	1st resolved n-alkane	SHWR	AWR	Naphthalene/TPAH ratio	TPAH (ppm)	TPH (ppm)
Pre-capping	1	13	1.019	1.082	0.013	333.700	109,000
	2	14	1.019	1.084	0.017	538.600	111,000
	3	18	1.000	1.150	0.029	0.011	1
	4	14	1.034	1.137	0.016	506.700	81,100
	5	13	1.026	1.099	0.013	655.100	121,000
	6	13	1.022	1.129	0.021	847.280	148,000
Post-capping	1	11	1.150	1.288	0.100	0.058	12
	2	14	1.490	2.611	0.406	0.004	4
	3	15	1.002	1.031	0.000	59.194	49,373
	4	ND	ND	1.388	0.179	0.001	ND
	5	11	1.133	1.267	0.145	0.012	2
	6	14	1.003	1.030	0.020	0.088	61
Pre-cap average		14	1.020	1.114	0.018	480	95,017
Post-cap average		13	1.156	1.436	0.142	10	9890
Standard dev pre-cap			0.011	0.029	0.006		
Standard dev post-cap			0.200	0.594	0.147		

*ND* not detected, *LMW* low molecular weight, *SHWR* saturated hydrocarbon weathering ratio, *AWR* aromatic weathering ratio, *TPAH* total polycyclic aromatic hydrocarbon, *TPH* total petroleum hydrocarbons

### GC-MS diagnostic ratios

Diagnostic ratios and critical difference analyses were performed on four of the six post-capping samples. In the first analysis of biomarker ratios, the post-capping samples 3 and 6 yielded positive results, and samples 2 and 4 yielded a probable result. Based on the weight of analytical evidence, it was evident that a probable result was yielded because the majority of the diagnostic ratios matched with the MC-252 reference samples. Detailed analytical results of diagnostic ratios are presented in Table 3.

**Table 3:** Diagnostic ratios of hopanes and steranes

Sample	Ts/Tm^a^	Ts/(Ts+Tm)	C29/C30	C31S/(S+R)	C32S/(S+R)	C33S/(S+R)	C34S/(S+R)	DR27bbR	DR28bbR	DR29bbR	DR27bb/29bb
MC-252 ref.	1.2 ± 0.10	0.54 ± 0.02	0.48 ± 0.03	0.60 ± 0.01	0.59 ± 0.01	0.59 ± 0.01	0.58 ± 0.01	0.72 ± 0.02	0.35 ± 0.02	0.48 ± 0.02	1.29 ± 0.05
NIST 2779	1.2 ± 0.08	0.54 ± 0.02	0.49 ± 0.01	0.59 ± 0.01	0.60 ± 0.01	0.57 ± 0.01	NA	0.72 ± 0.01	0.37 ± 0.01	0.46 ± 0.01	1.33 ± 0.02
Sample 2	1.15	0.53	0.92	0.54	0.57	0.51	0.47	0.63	0.31	0.61	1.03
Sample 3	1.09	0.52	0.54	0.59	0.60	0.60	0.60	0.73	0.40	0.41	1.45
Sample 4	0.91	0.48	0.65	0.56	0.62	0.61	0.57	0.39	0.36	0.84	0.61
Sample 6	1.03	0.51	0.56	0.59	0.57	0.59	0.58	0.62	0.39	0.50	1.15
	Diff	Diff	Diff	Diff	Diff	Diff	Diff	Diff	Diff	Diff	Diff
Sample 2	0.05	0.01	−0.43	0.05	0.02	0.08	0.11	0.09	0.04	−0.13	0.26
Sample 3	0.11	0.02	−0.05	0.00	−0.01	−0.01	−0.02	−0.01	−0.05	0.07	−0.16
Sample 4	0.29	0.06	−0.16	0.03	−0.03	−0.02	0.01	0.33	−0.01	−0.36	0.68
Sample 6	0.17	0.03	−0.07	0.00	0.02	0.00	0.00	0.10	−0.04	−0.02	0.14
	Mean	Mean	Mean	Mean	Mean	Mean	Mean	Mean	Mean	Mean	Mean
Sample 2	1.17	0.54	0.71	0.57	0.58	0.55	0.52	0.68	0.33	0.54	1.16
Sample 3	1.15	0.53	0.52	0.59	0.59	0.59	0.59	0.73	0.37	0.45	1.37
Sample 4	1.06	0.51	0.57	0.58	0.60	0.60	0.57	0.55	0.36	0.66	0.95
Sample 6	1.12	0.52	0.52	0.59	0.58	0.59	0.58	0.67	0.37	0.49	1.22
	Rel diff %	Rel diff %	Rel diff %	Rel diff %	Rel diff %	Rel diff %	Rel diff %	Rel diff %	Rel diff %	Rel diff %	Rel diff %
Sample 2	4.35	1.00	−61.16	7.97	2.80	14.63	21.32	12.91	13.17	−23.38	22.81
Sample 3	9.17	3.27	−10.01	−0.71	−1.05	−1.08	−3.56	−1.91	−13.18	15.46	−11.87
Sample 4	26.97	12.23	−27.79	5.04	−4.82	−3.91	2.19	60.50	−3.71	−54.08	71.41
Sample 6	15.04	6.13	−12.98	0.46	2.75	−0.62	0.49	14.37	−11.76	−4.14	11.33
	Concl	Concl	Concl	Concl	Concl	Concl	Concl	Concl	Concl	Concl	Concl
Sample 2	M	M	NM	M	M	M	NM	M	M	NM	NM
Sample 3	M	M	M	M	M	M	M	M	M	NM	M
Sample 4	NM	M	NM	M	M	M	M	NM	M	NM	NM
Sample 6	M	M	M	M	M	M	M	M	M	M	M

Forensic analysis results, showing diagnostic ratios and critical difference calculations. Level of association between environmental samples and the MC-252 reference samples shown.

MC-252 ref. Deepwater Horizon reference sample; NIST 2779 National Institute of Standards and Technology: Standard Reference Material, Gulf of Mexico Crude Oil (SRM 2779) MC-252; *Diff* difference, *Rel Diff%* percent relative difference, *Concl* conclusion, *M* match, *NM* no match

^a^A description of the diagnostic ratios is provided in Table 1 of the Online Resource supplemental information link

## Discussion

Biomarker analysis of post-capping samples suggests that the MC-252 well was the source of the oil found in the coastal and offshore field samples. Post-capping samples 2 and 4 yielded a probable correlation, based upon the weight of evidence, and are similar to MC-252 oil. These samples yielded the lowest concentration of hydrocarbons within the entire dataset, resulting in weaker biomarker responses. Hopane peak height responses for samples 2 and 4 were 216 and 4126 respectively, compared to 577,691 and 1,180,545 for samples 3 and 6, respectively. All of our biomarker ratios were consistent with a match for both MC-252 reference samples and the NIST SRM 2779 sample. It should be noted that certain aspects of analytical processing, such as capillary columns, operating temperatures, and mass spectrometer dwell times, can affect biomarker ratios (CEN 2011) and should be taken into consideration. It is also known that environmental samples can contain co-extractives which can affect biomarker ratios (Aeppli et al. 2014). We followed the procedure of CEN/TR 15522-2:2012 which stipulates column requirements, operating conditions including temperature ranges and mass spectrometer dwell times. Furthermore, proper operating conditions were checked by analyzing reference oil provided by SINTEF Materials and Chemistry, Trondheim, Norway.

Analysis of n-alkane and PAH concentrations showed that four of the post-capping samples were less weathered than the six pre-capping samples. Some of our post-capping samples appear to be as fresh as those discussed in the literature. Liu et al. (2012) collected emulsified oil samples during the spill period (May 2010), and they found resolved n-alkanes ≥ C_14_ compared to our post-capping samples which had an average of C_13_ n-alkanes.

Other discussions of the post-capping leaks suggest that the source of oil slicks in 2011 and 2012 is from the submerged *Deepwater Horizon* rig, e.g., small quantities (∼1 bbl day^−1^) of oil leaking from the stranded cofferdam or pipeline riser (Dittrick 2012a, b; Aeppli et al. 2013). Other researchers suggest fresh post-capping MC-252 tarballs found on the beach are from portions of subtidal near-shore oil mats that were formed during the spill that wash ashore during severe weather events (Hayworth et al. 2011; OSAT-2 2011; Clement et al. 2012; Hayworth and Clement 2012; Mulabagal et al. 2013).

On three occasions we observed emulsified oil slicks 60 km NW from the MC-252 well site and 15 km offshore which appeared to have been floating on the ocean surface for 8 to 10 days. On two occasions, we observed large oil slicks 200 km from MC-252 and 18 km offshore. These oil slicks were weathered and sometimes finely dispersed and appeared to have spent about 30 days at sea. For those slicks to survive on the surface for that long and travel that far, the discharge had to be large, according to the dispersion model, >5,000 bbl d^−1^ (MMS 2000). Secondly, the oil we encountered was emulsified as indicated by the red-brown, brown, or beige color. This is an unusual color for a common spill of ≤50 bbl day^−1^ of Louisiana sweet crude suggesting it was from a large discharge. Photographs of the oil slicks observed during the study period are presented in the Field Observation section in the Online Resource supplement information link.

The post-capping oil slicks did not appear as widespread, persistent, or abundant as during the spill period; however, large waves of red-brown oil were observed intermittently during 2011 and 2012. It is possible that stranded equipment could have been discharging small quantities of oil (∼1 bbl day^−1^) that would produce a limited surface expression near the well site that would last ∼24 h on the surface and would not emulsify. Discharges from stranded equipment or near-shore tar mats cannot explain the field observations of emulsified oil slicks 60 to 200 km from the MC-252 well site and 18 to 20 km offshore.

The most probable source of the fresh oil was a ruptured well casing. The pressurized oil and gas will seek perforations in the adjacent geologic formations. It is not uncommon for a permanently plugged well to leak, especially one that is experiencing 10,000 psi of sustained casing pressure (Barclay et al. 2001; Cavanagh et al. 2007; Leifer and Wilson 2007; McNutt et al. 2012b). The initial drilling or the drilling of the relief wells could have damaged the well casing and/or the geologic formations adjacent to the well. Hydrocarbons could be escaping through ruptured seeps adjacent to the MC-252 well and through perforations. Oil and gas could have expanded into horizontal pathways until it intersected with an existing vertical fault and ascended to the seafloor. The presence of fresh MC-252 crude oil on the offshore water surface in the northern Gulf of Mexico, a year and 10 months after capping, suggests that the oil and gas from the MC-252 field was leaking during the study period.

## Electronic supplementary material

ESM 1 (PDF 2.13 MB)
