# Supplementary material for: Oil in the Gulf of Mexico after the capping of the BP/Deepwater Horizon Mississippi Canyon (MC-252) well
Source: Environ Sci Pollut Res Int. 2015 Apr 16;22(16):12073–82. doi: 10.1007/s11356-015-4421-y (PMC4515244; doi:10.1007/s11356-015-4421-y)
Supplement: Supplementary file 1 — (PDF 2.13 MB) [file 11356_2015_4421_MOESM1_ESM.pdf]

ONLINE RESOURCE

SUPPLIMENTAL INFORMATION

OIL IN THE GULF OF MEXICO AFTER THE CAPPING OF THE BP/DEEPWATER HORIZON MISSISSIPPI CANYON (MC-252) WELL

Published by

Environmental Science and Pollution Research

Prepared by

Steve R. Kolian[a,*], Scott A. Porter [a], Paul W. Sammarco[a,b], Detlef Birkholz[c],
Edwin Cake[d], and Wilma Subra[e]

[*]Corresponding author:

[a]EcoRigs Non-Profit Organization
6765 Corporate Blvd., Suite 1207, Baton Rouge, LA 70809 USA
stevekolian@ecorigs.org

[b]Louisiana Universities Marine Consortium (LUMCON)
8124 Hwy. 56, Chauvin, LA 70344 USA

[c]ALS Environmental
5424 – 97 Street, Edmonton, AB T6E 5C1, Canada
deib.birkholz@alsglobal.com

[d]Gulf Environmental Associates,
2510 Ridgewood Road, Ocean Springs, MS 39564 USA

[e]Louisiana Environmental Action Network (LEAN), and
Lower Mississippi Riverkeepers
PO Box 9813, New Iberia, LA 70562 USA

2015

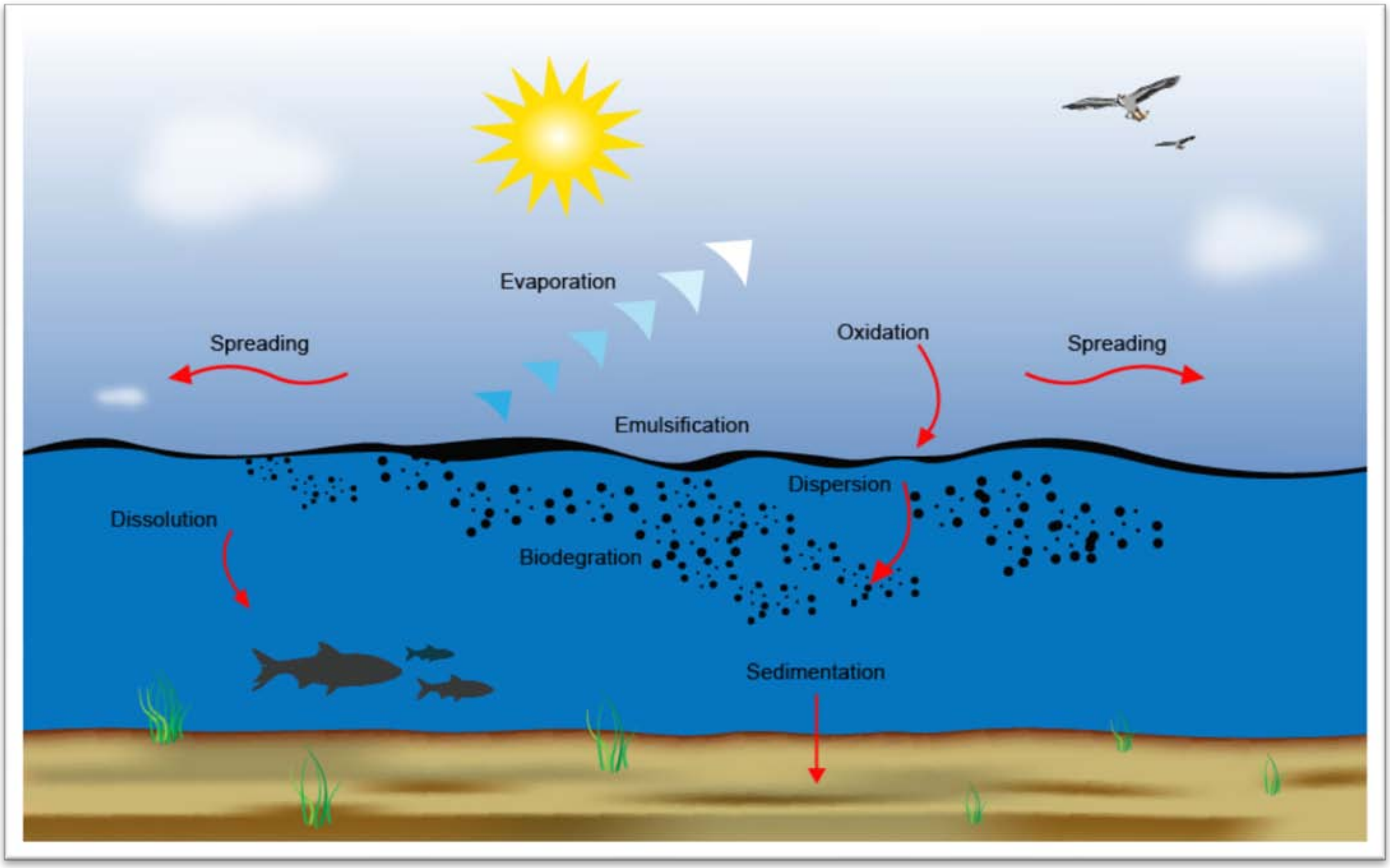

Fig. 1 Oil slicks weather by spreading, evaporation, emulsification, dispersion, sedimentation, and photo and microbial oxidation. Oil slicks quickly spread to cover extensive areas of the sea surface. Dispersion occurs when oil is broken up into small globules (50 to 400 μm) that eventually descend through the water column via digestion by bacteria or they attach to carbon or sediments and sink into the sediments (CEN 2011).

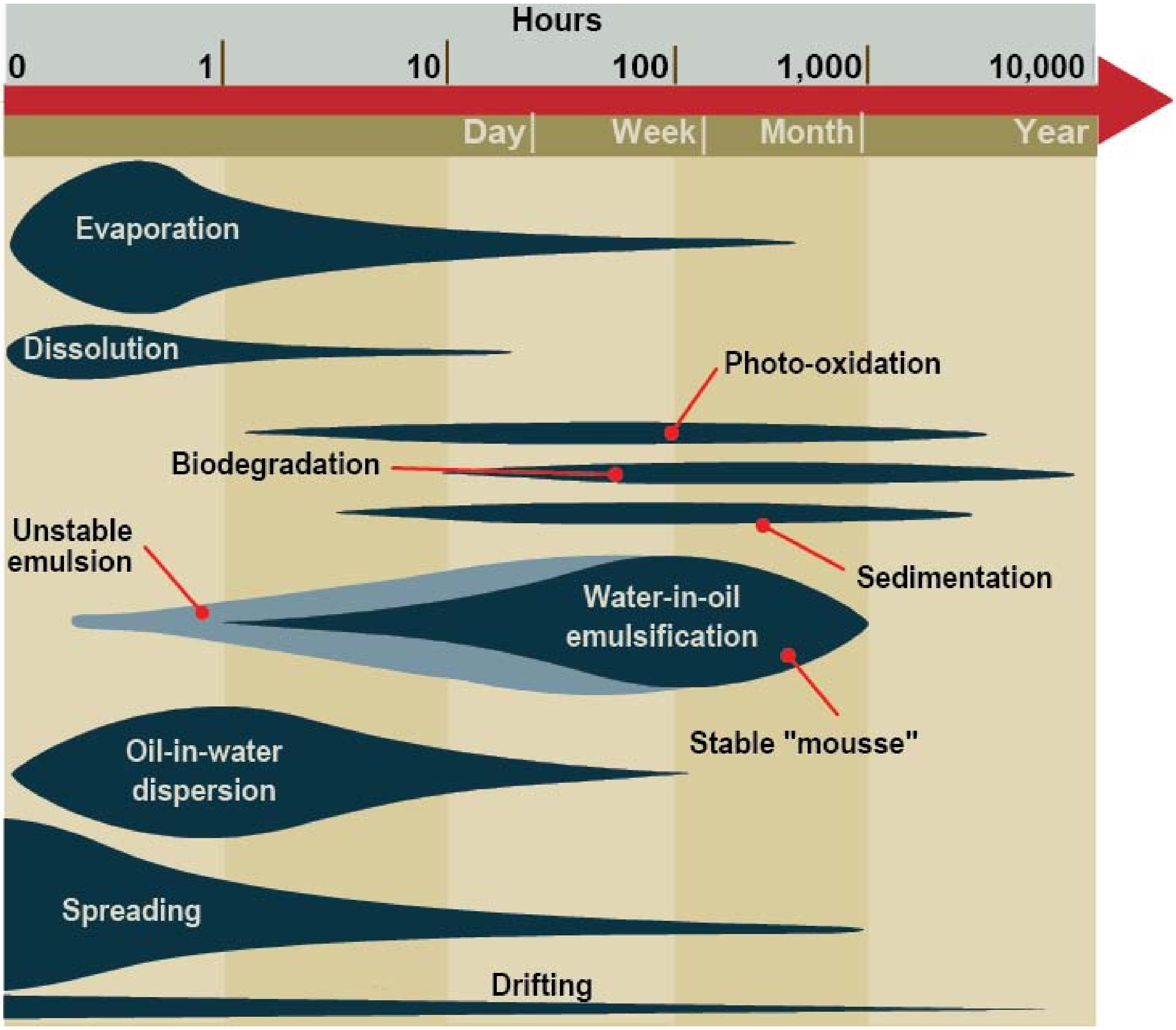

Fig. 2 Weathering timeline of oil spills. A large spill of viscous oil can emulsify and remain on the surface ≥ 30 days before the forces of dispersion or sedimentation overcome the forces of emulsion and sink the oil (ITOPF 2002; Daling 2011). Tar balls are composed of oil and sediment, neutrally buoyant, and are known to float around in the region of the spill for 6 to 9 months.

Compound	Biomarker Ratio
**Hopanes**	Ts/Tm
	Ts/(Ts+Tm)
	C29/C30
	C31S/(S+R)
	C32S/(S+R)
	C33S/(S+R)
	C34S/(S+R)
**Steranes**	DR27bbR = 27bb(S+R)/28bb(S+R) + 29bb(S+R)
	DR28bbR = 28bb(S+R)/ 27bb(S+R) + 29bb(S+R)
	DR29bbR = 29bb(S+R)/27bb(S+R) + 28bb(S+R)
	DR27bb/29bb = 27bb(S+R)/29bb(S+R)

Table 1 Biomarker ratios used in diagnostic analysis.

Pre-Capping PAHs (ppm)	Sample 1	Sample 2	Sample 3	Sample 4	Sample 5	Sample 6
Sample Date	23-May-10	23-May-10	24-May-10	6-Jun-10	7-Jun-10	7-Jun-10
1-Methylnaphthalene	<1.0	<1.0	<0.00002	<1.0	<1.0	<1.0
2-Methylnaphthalene	<1.0	<1.0	<0.00002	<1.0	<1.0	<1.0
Acenaphthene	<1.0	<1.0	<0.00002	<1.0	<1.0	<1.0
Acenaphthylene	<1.0	<1.0	<0.00002	<1.0	<1.0	<1.0
Acridine	<1.0	<1.0	<0.00002	<1.0	<1.0	<1.0
Anthracene	<1.0	<1.0	<0.00002	<1.0	<1.0	<1.0
Benz(a)anthracene	<1.0	<1.0	<0.00002	<1.0	<1.0	<1.0
Benzo(a)pyrene	<1.0	<1.0	<0.00002	<1.0	<1.0	<1.0
Benzo(b&j)fluoranthene	<1.0	1.5	0.000022	<1.0	1.4	1.7
Benzo(e)pyrene	1.3	2.0	0.000028	1.2	1.8	2.1
Benzo(g,h,i)perylene	<1.0	<1.0	<0.00002	<1.0	<1.0	<1.0
Benzo(k)fluoranthene	<1.0	<1.0	<0.00002	<1.0	<1.0	<1.0
Biphenyl	<1.0	<1.0	<0.00002	<1.0	<1.0	<1.0
C1 Acenaphthenes	<4.0	<4.0	<0.00008	<4.0	<4.0	<4.0
C1 Benz(a)Anthracenes/Chrysenes	16.7	27.0	0.000392	19.6	29.8	37.9
C1 Benzofluoranthenes/Benzopyrenes	4.7	6.9	0.000143	4.5	6.8	8.1
C1 Biphenyls	<4.0	<4.0	<0.00008	<4.0	<4.0	<4.0
C1 Dibenzothiophenes	4.1	6.1	0.000234	8.1	10.6	13.6
C1 Fluoranthenes/Pyrenes	<4.0	4.7	<0.00008	4.1	4.6	6.0
C1 Fluorenes	<1.0	<1.0	0.000129	4.2	<1.0	5.6
C1 Phenanthrenes/Anthracenes	30.7	46.9	0.00145	56.1	65.8	89
C2 Benz(a)Anthracenes/Chrysenes	<1.0	<1.0	0.00031	<1.0	<1.0	<1.0
C2 Benzofluoranthenes/Benzopyrenes	<4.0	5.1	<0.00008	4.6	5.7	7.5
C2 Biphenyls	<4.0	<4.0	<0.00008	<4.0	<4.0	<4.0
C2 Dibenzothiophenes	12.3	18.5	0.000532	21.1	26.6	37.3
C2 Fluoranthenes/Pyrenes	<4.0	5.7	0.000132	6.2	8.0	9.6
C2 Fluorenes	5.5	8.0	0.00029	13.2	13.7	18.9
C2 Naphthalenes	<1.0	<1.0	<0.00002	<1.0	<1.0	<1.0
C2 Phenanthrenes/Anthracenes	63.1	95.7	0.00188	86.4	112	144
C2 sub'd B(a)A/chrysene	15.1	23.4	<0.00002	16.7	26.4	31.4
C3 Benzanthracenes/Chrysenes	5.8	7.6	0.000112	7.2	13.4	15.5
C3 Dibenzothiophenes	12.3	18.7	0.000471	20.6	25.7	32.0
C3 Fluoranthenes/Pyrenes	<4.0	6.0	0.000086	9.1	13.1	15.8
C3 Fluorenes	10.8	15.5	0.000429	22.8	22.9	33.2
C3 Naphthalenes	<1.0	<1.0	0.000167	<1.0	<1.0	4.2
C3 Phenanthrenes/Anthracenes	40.3	70.9	0.000984	58.6	73.2	93.4
C4 Benzanthracenes/Chrysenes	<4.0	5.4	<0.00008	5.3	7.0	10.1
C4 Dibenzothiophenes	7.1	9.3	0.000409	11.5	17.9	17.8
C4 Fluoranthenes/Pyrenes	6.9	9.9	0.000142	10.7	16.3	19.4
C4 Naphthalenes	4.4	8.9	0.000146	8.3	8.6	13.2
C4 Phenanthrenes/Anthracenes	79.8	114	0.00169	84.4	116	142
Chrysene	8.9	14.7	0.00024	12.1	18.3	23.0
Dibenz(a,h)anthracene	<1.0	<1.0	<0.00002	<1.0	<1.0	<1.0
Dibenzothiophene	<1.0	<1.0	0.00004	<1.0	<1.0	1.1
Fluoranthene	<1.0	<1.0	<0.00002	<1.0	<1.0	<1.0
Fluorene	<1.0	<1.0	0.000026	<1.0	<1.0	<1.0
Indeno(1,2,3-cd)pyrene	<1.0	<1.0	<0.00002	<1.0	<1.0	<1.0
Naphthalene	<1.0	<1.0	<0.00002	<1.0	<1.0	<1.0
Perylene	<1.0	<1.0	<0.00002	<1.0	<1.0	<1.0
Phenanthrene	2.6	4.2	0.000232	6.9	7.5	10.4
Pyrene	<1.0	<1.0	<0.00002	<1.0	<1.0	<1.0
Quinoline	<1.0	<1.0	<0.00002	<1.0	<1.0	<1.0
Retene	1.3	2	0.000023	3.2	2	3.5
Total PAH	333.7	538.6	0.010739	506.7	655.1	847.3

Table 2. Concentrations (ppm) of PAHs and Alkylated PAHs in pre-capping environmental samples.

Post-Capping PAHs (ppm)	Sample 1	Sample 2	Sample 3	Sample 4	Sample 5	Sample 6
Sample Date	12-Sep-10	12-Sep-10	28-Mar-11	3-Apr-11	16-Aug-11	22-May-12
1-Methylnaphthalene	<0.000010	0.000082	<0.02	<0.000010	0.000092	0.000049
2-Methylnaphthalene	<0.000010	0.000173	<0.02	<0.000010	0.000012	0.000082
Acenaphthene	0.000020	0.000024	<0.02	<0.000010	<0.000010	<0.000010
Acenaphthylene	0.000019	<0.000010	<0.02	<0.000010	<0.000010	<0.000010
Acridine	0.000026	<0.000010	<0.02	<0.000010	<0.000010	<0.000010
Anthracene	<0.000010	0.000023	<0.02	<0.000010	<0.000010	<0.000010
Benz(a)anthracene	0.000054	<0.000010	<0.02	<0.000010	0.000018	<0.000010
Benzo(a)pyrene	0.000013	<0.000010	<0.02	<0.000010	0.000020	<0.000010
Benzo(b&j)fluoranthene	0.000049	<0.000010	<0.02	<0.000010	0.000017	<0.000010
Benzo(e)pyrene	0.000118	<0.000010	0.84	<0.000010	0.000034	<0.000010
Benzo(g,h,i)perylene	0.000012	<0.000010	0.07	<0.000010	0.000027	<0.000010
Benzo(k)fluoranthene	0.00001	<0.000010	<0.02	<0.000010	0.000028	<0.000010
Biphenyl	0.000012	0.000056	<0.02	<0.000010	0.000032	0.000012
C1 Acenaphthenes	0.000094	<0.000010	<0.02	<0.000010	<0.000010	<0.000010
C1 Benz(a)Anthracenes/Chrysenes	0.00168	<0.000010	5.35	<0.000010	0.000240	0.002820
C1 Benzofluoranthenes/Benzopyrenes	0.000476	<0.000010	2.70	<0.000010	0.000152	0.001160
C1 Biphenyls	<0.000040	0.000094	<0.02	<0.000010	<0.000010	<0.000010
C1 Dibenzothiophenes	0.000187	<0.000010	0.19	<0.000010	0.000086	0.000148
C1 Fluoranthenes/Pyrenes	0.000557	<0.000010	0.71	<0.000010	0.000102	0.000480
C1 Fluorenes	0.000065	0.000126	<0.02	<0.000010	0.000041	0.000090
C1 Phenanthrenes/Anthracenes	0.000721	0.000320	<0.02	0.000077	0.000606	0.001710
C2 Benz(a)Anthracenes/Chrysenes	0.00267	<0.000010	<0.02	<0.000010	0.000321	0.003070
C2 Benzofluoranthenes/Benzopyrenes	0.000834	<0.000010	2.82	<0.000010	0.000126	0.001300
C2 Biphenyls	0.00032	0.000278	<0.080	<0.000010	0.000078	0.000058
C2 Dibenzothiophenes	0.00139	<0.000010	0.71	<0.000010	0.000240	0.002010
C2 Fluoranthenes/Pyrenes	0.00133	<0.000010	1.22	<0.000010	0.000154	0.001000
C2 Fluorenes	0.00116	<0.000010	<0.02	<0.000010	0.000113	0.000198
C2 Naphthalenes	0.00008	0.000393	<0.02	<0.000010	0.000609	0.000279
C2 Phenanthrenes/Anthracenes	0.00624	0.000375	3.98	0.000146	0.001870	0.015700
C2 sub'd B(a)A/chrysene	<0.000010	<0.000010	5.18	<0.000010	<0.000010	<0.000010
C3 Benzanthracenes/Chrysenes	0.00199	<0.000010	3.31	<0.000010	0.0000199	0.002020
C3 Dibenzothiophenes	0.00192	<0.000010	1.24	<0.000010	0.000347	0.004590
C3 Fluoranthenes/Pyrenes	0.00189	<0.000010	1.95	<0.000010	0.000017	0.000861
C3 Fluorenes	0.00242	0.000119	0.76	0.000056	0.000212	<0.000010
C3 Naphthalenes	0.00121	0.000522	<0.02	0.000041	0.000685	0.000415
C3 Phenanthrenes/Anthracenes	0.007480	0.000202	7.43	0.000084	0.002030	0.002330
C4 Benzanthracenes/Chrysenes	0.000709	<0.000010	2.26	<0.000010	0.000071	0.001700
C4 Dibenzothiophenes	0.001280	<0.000010	1.60	<0.000010	0.000278	0.004060
C4 Fluoranthenes/Pyrenes	0.001710	<0.000010	3.43	<0.000010	0.000170	0.002380
C4 Naphthalenes	0.004510	0.000474	<0.02	0.000065	0.000487	0.000405
C4 Phenanthrenes/Anthracenes	0.013600	<0.000010	9.75	0.00011	0.002630	0.037700
Chrysene	0.000280	<0.000010	3.30	<0.000010	0.000029	0.000291
Dibenz(a,h)anthracene	0.000022	<0.000010	<0.02	<0.000010	0.000012	<0.000010
Dibenzothiophene	0.000029	0.000028	<0.02	<0.000010	0.000021	0.000019
Fluoranthene	<0.000010	0.000059	<0.02	<0.000010	0.000014	<0.000010
Fluorene	0.000026	0.000055	<0.02	<0.000010	0.000017	0.000047
Indeno(1,2,3-cd)pyrene	<0.000010	<0.000010	<0.02	<0.000010	0.000011	<0.000010
Naphthalene	<0.000010	0.00023	<0.02	<0.000010	<0.000010	0.00065
Perylene	0.000435	<0.000010	0.06	<0.000010	0.00001	<0.000010
Phenanthrene	0.000012	0.000266	<0.02	<0.000010	<0.000010	0.000123
Pyrene	0.000047	0.000064	0.07	<0.000010	0.000024	<0.000010
Quinoline	<0.000010	<0.000010	<0.02	<0.000010	<0.000010	<0.000010
Retene	0.000428	0.000025	<0.02	0.000013	0.000205	<0.000010
Total PAH	0.058135	0.003988	58.93	0.000592	0.0123079	0.087757

Table 3. Concentrations (ppm) of PAHs and Alkylated PAHs in post-capping environmental samples.

COMPARISON OF MC-252 TO OTHER COMMON DISCHARGES IN THE GULF OF MEXICO

Small discharges of crude oil from offshore platforms, pipelines, and natural sources are common in the federal waters of the Gulf of Mexico (National Response Center 2014; MacDonald *et al.* 1993, 1996); however, large discharges >5,000 bbl d^{-1} are rare (Eschenbach *et al.* 2010). The MC-252 oil spill changed color from other common spills in the region because the discharge rate was significantly greater and it was released 1,500 m below the ocean surface.

The majority of the oil produced in the northern Gulf of Mexico is Louisiana sweet crude, a type of oil that favors dispersion over emulsification (MMS 2000). When seawater mixes into oil with greater viscosities, emulsification will occur. Emulsified oil has pudding like texture and is often called mousse, referring to chocolate mousse. The viscous buoyant properties of emulsified oil counter the forces of dispersion and the slick stabilizes which can cause the oil to drift in the currents for extended periods. When oil starts to emulsify, it turns red and then rusty-red and progresses to brown-beige color (Belore *et al.* 2011; Daling 2011) and eventually particulates and disperses into the water column in ~30 days in calm seas (API 1999; ITOPF 2002; CEN 2011). Only 14% of the oil in the region are highly viscous and favor emulsification (MMS 2000).

Most oil spills in the region are <50 bbl d^{-1} and they have a violate, metallic, or rainbow color and only last ≤72 hours on the surface of the ocean before dispersing. Out of the well, the MC-252 crude, like most oils in the region, had a 37° API gravity and a low 4 centipoise [cP] viscosity (Belore *et al.* 2011; Daling 2011) indicating that it would naturally disperse relatively fast and not form an emulsion (MMS 2000); however, Belore *et al.* (2011) and Ryerson *et al*. (2011) noted that the MC-252 oil started to form a water-in-oil emulsion during its ascent to the surface as the plume lost its C_1 to C_9 compounds. The viscosity increased from 4 cP exiting the well to 1,000 cP after a few hours on the ocean surface and viscosity continued to up to 10,000 cP as the C_{10} to C_{13} volatile compounds evaporated and the oil absorbed water (Daling 2011). The MC-252 oil formed a stable water-in-oil emulsion mousse in ~48 hours (Belore *et al.* 2011; Reddy *et al.* 2012; Ryerson *et al.* 2012). Therefore, if there is emulsified red-brown or brown or beige oil in offshore waters, the probability of it being from MC-252 well is high. All the sightings and photographs of oil in the following section are emulsified oil or from sheens adjacent to emulsified oil.

FIELD OBSERVATIONS

The following contains information and observations collected during routine vessel research trips. A brief narrative, photographs, and a location map of some of the oil sightings are presented in the following pages. The location and photographs of samples collected and other observations of oil slicks during the study period are presented in chronological order.

FIG. 2 Observation Date – 12 September 2010

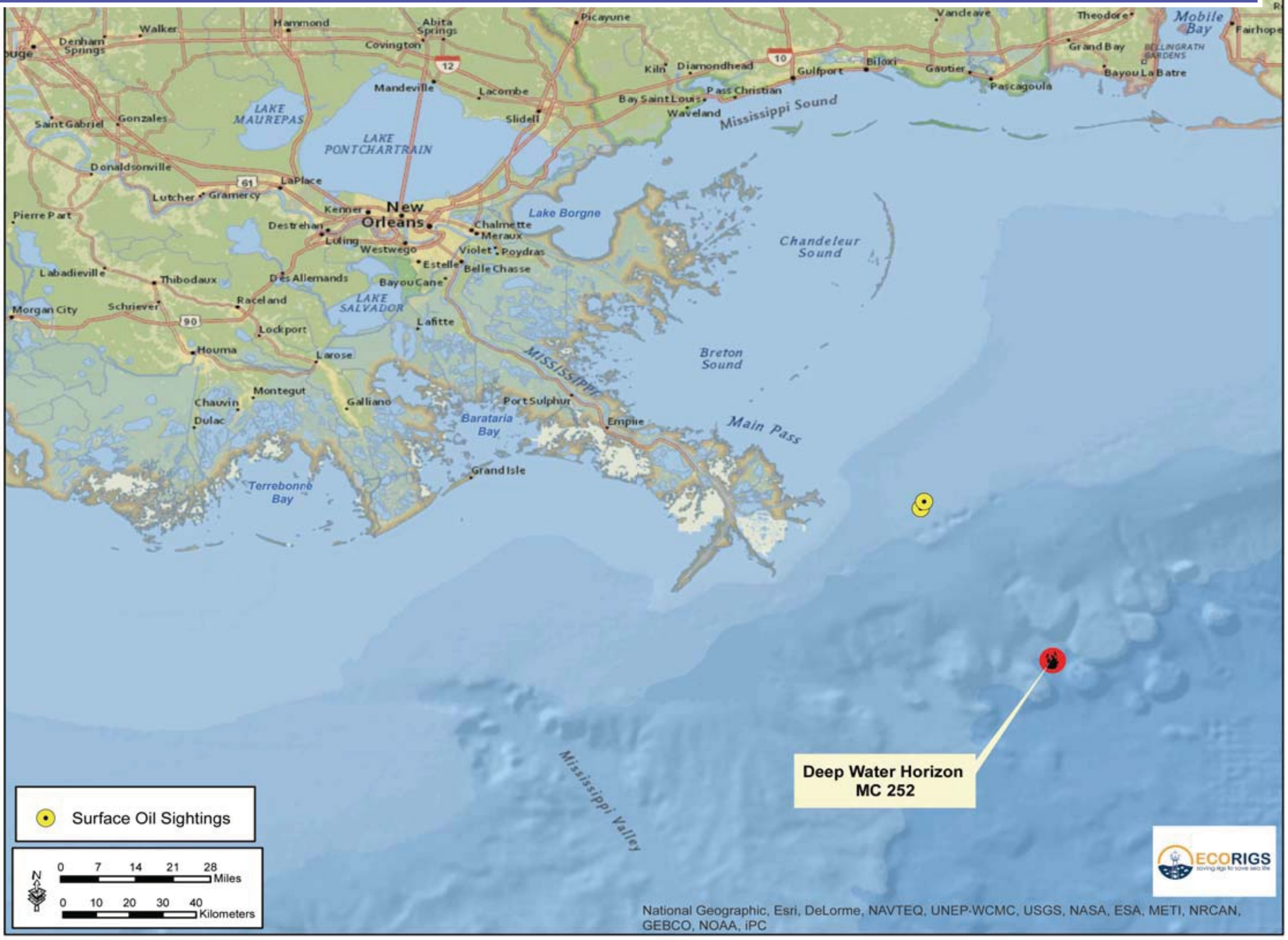

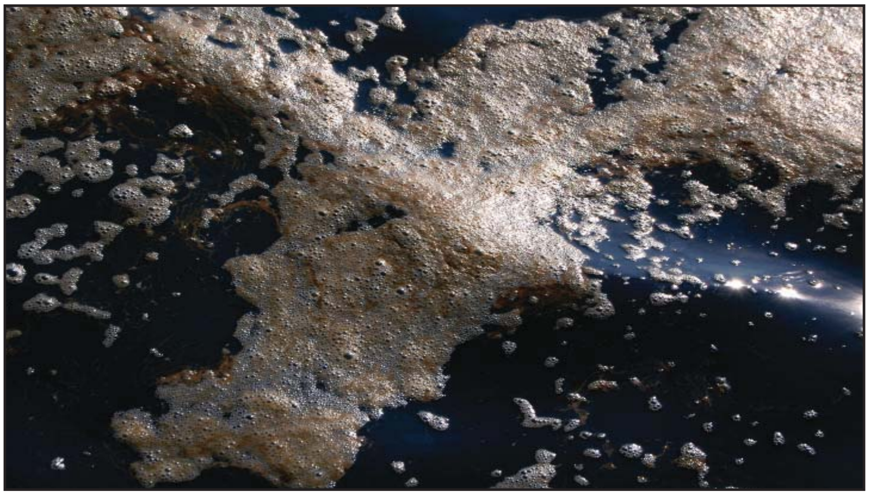

◄ The first oil slick sample in this study was collected 59 days (12 September 2012) after the capping of MC-252 blowout well near an offshore platform Main Pass (MP) 311. Forensics analysis of the crude oil confirmed MC-252 as its source.

Observation Date – 12 September 2010 (continued)

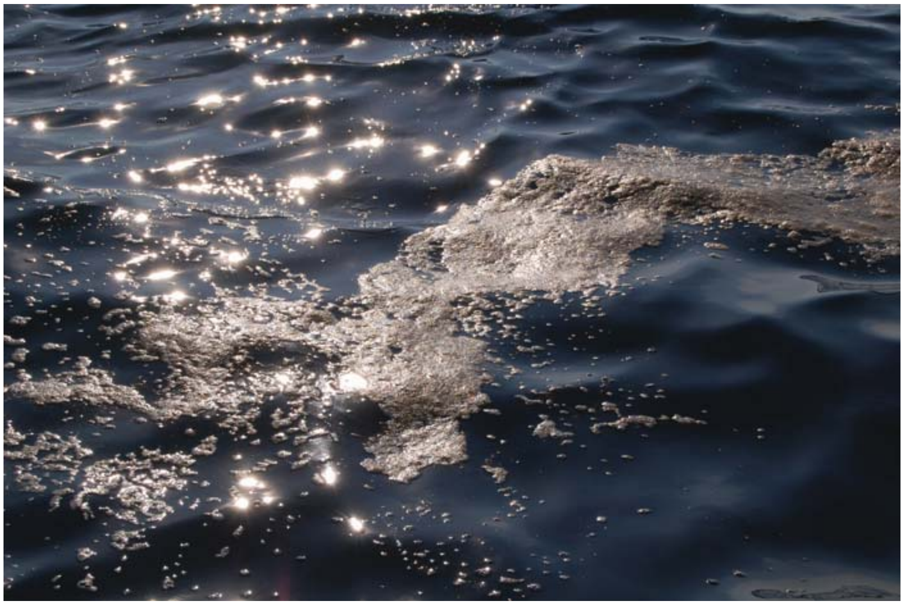

◄ From the vessel, waves of foamy oil were observed during a 3 hour reserearch event in the area. Two distinct types of oil slicks were observed frem the vessel. There were waves of foamy brown emulsified oil and violet sheen.

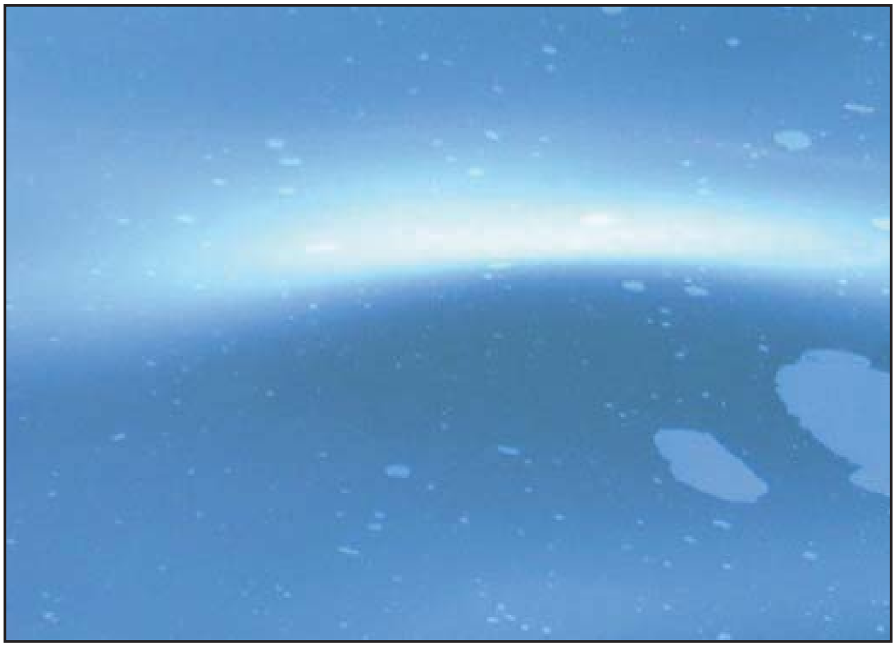

◄ The ocean surface contained intermittent waves of a translucent violet sheen that appeared to be spreading from the brown crude oil, which appeared to be weathered and the sheen appeared fresh and contained thin drops of volitile rainbow colored oil. This processes is called "sheening" when remnaining LMW compounds escape.

Observation Date – 12 September 2010 (continued)

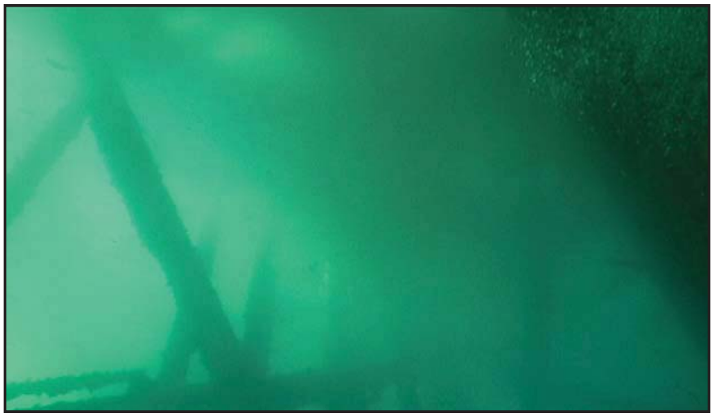

◄ Research divers documented subsurface plumes of dispersed oil at MP-311. Cloud-like plumes of subsurface dispersed crude oil drifted through the structure intermittently during the dive. The divers were visiting the platform to collect marine invertabrates and found visible subsurface plumes.

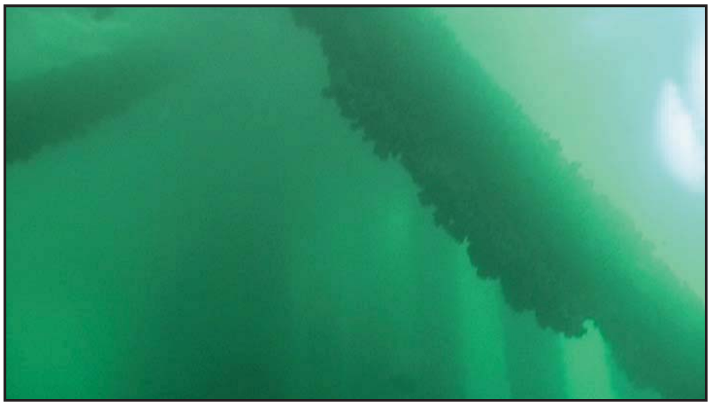

◄ ▼ The plumes of dispersed oil observed in the upper 10 m of the water column. Marine invertebrates were collected and sent to the lab and analyzed for total petroleum hydrocarbons (TPHs). Concentrations ranged from 210 to 2,300 ppm. The highest concentrations of TPHs were found in the heterotrophic corals *Tabastrea coccinea*. (Sammarco et al. 2013).

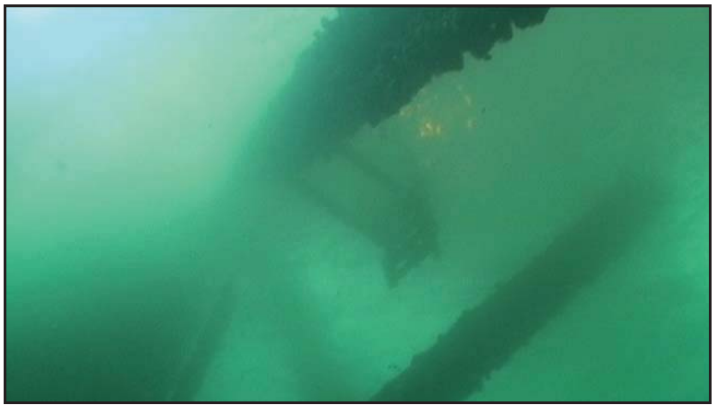

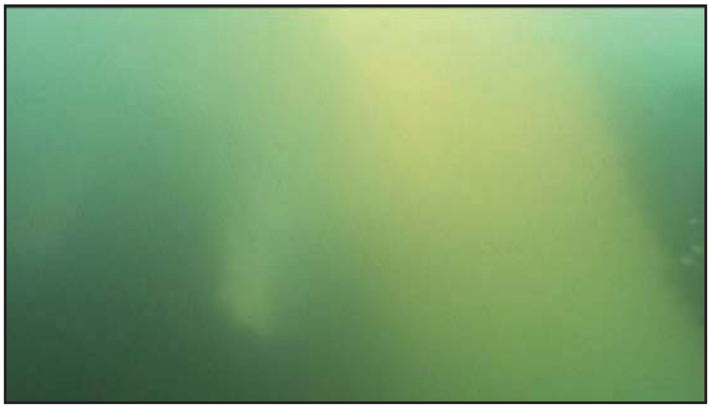

FIG. 3 Observation Date – 3 April 2011

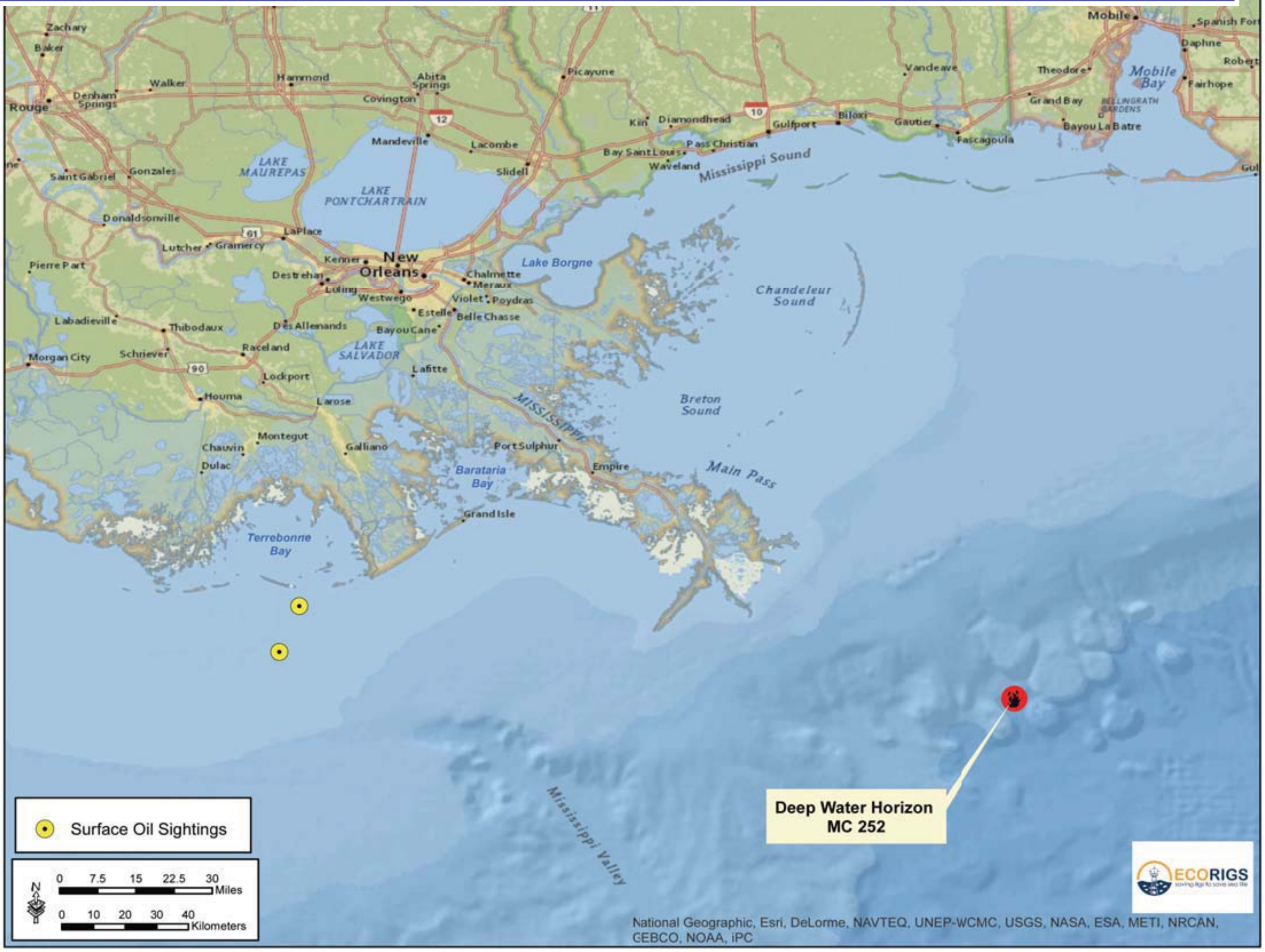

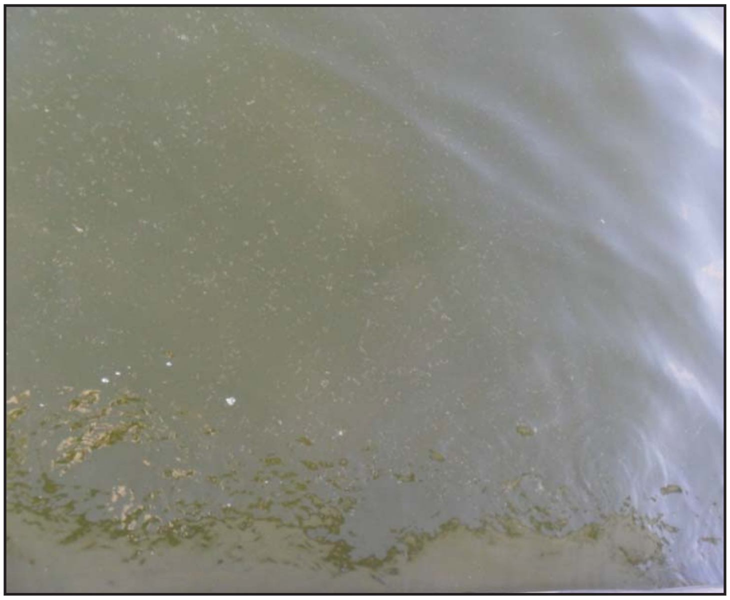

◄ Oil was observed south 10 to 20 km of of Timbalier Island and 50 km west of Port Fourchon, Louisiana. The oil appeared to be in an advanced state of weathering as it was observed to break into smaller particles.

Observation Date – 3 April 2011 (continued)

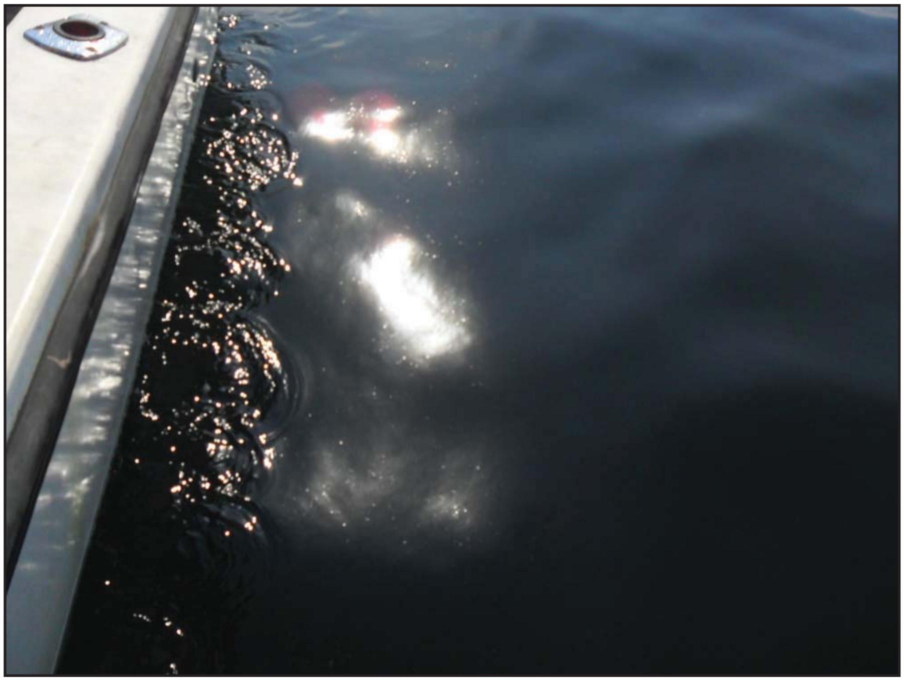

◄ The crude oil was present over a 6 km wide area, flowing parallel to the coast from east to west. Water sampling and subsequent analysis confirmed the oil originated from the MC-252 field. An oil sheen was observed on the surface and strands of emulsified oil appeared below the surface.

FIG. 4 Observation Date – 16 August 2011

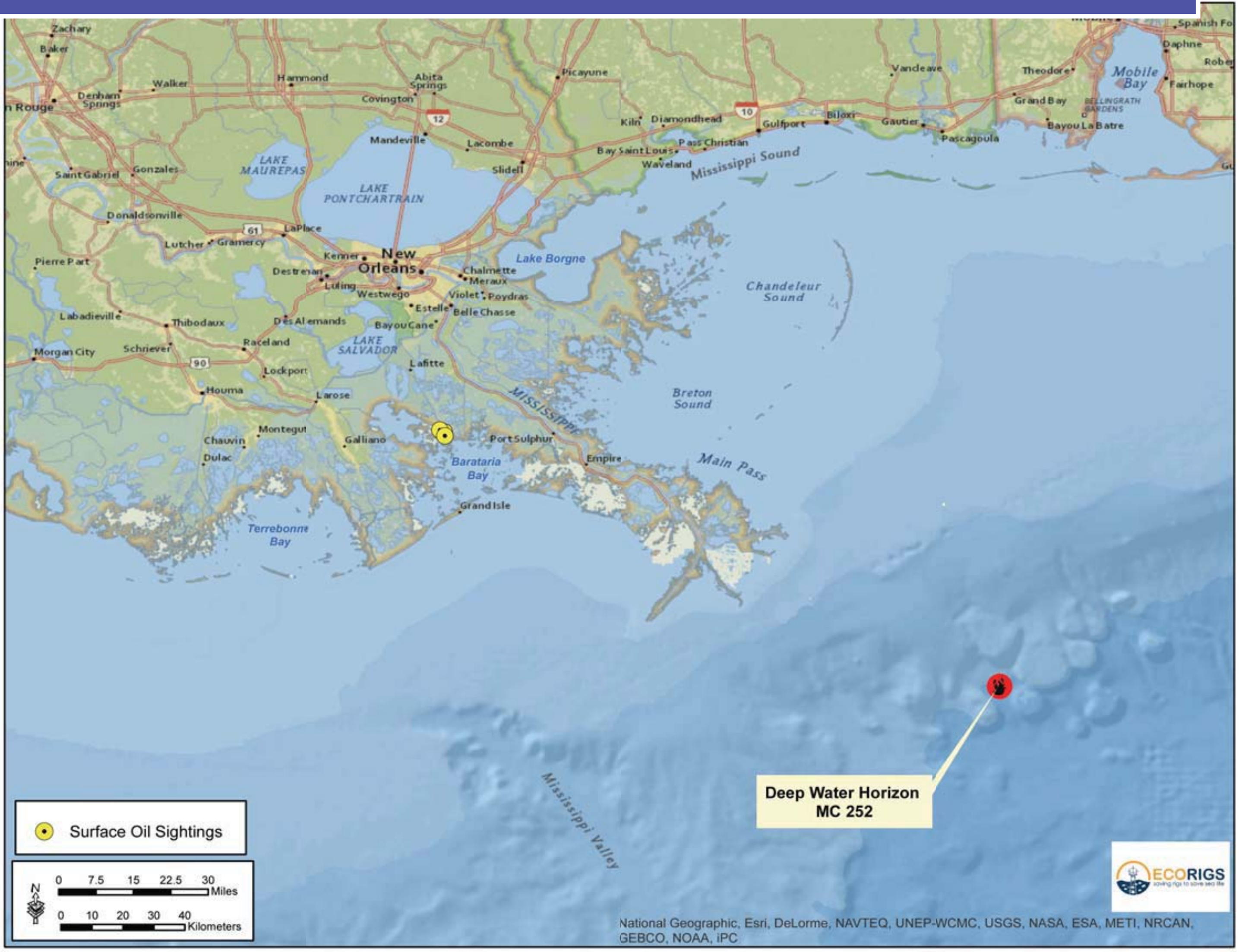

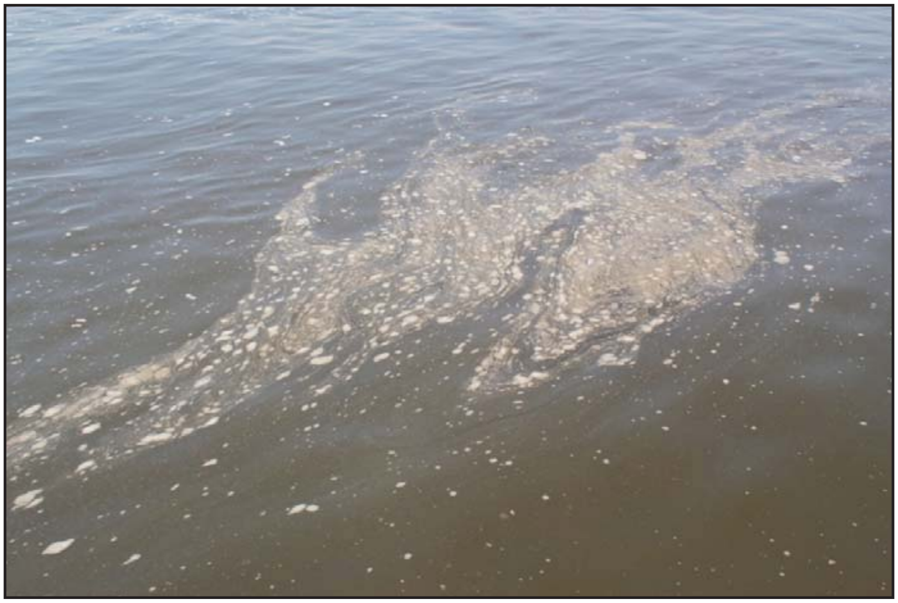

◄ The surface oil was observed by vessel 10 km upstream of Barataria Bay in Bayou Saint Denis, Louisiana on 16 August 2011. Bayou Saint Denis was filled with thick brown patches of surface oil and partially submerged emulsified oil.

Observation Date – 16 August 2011 (continued)

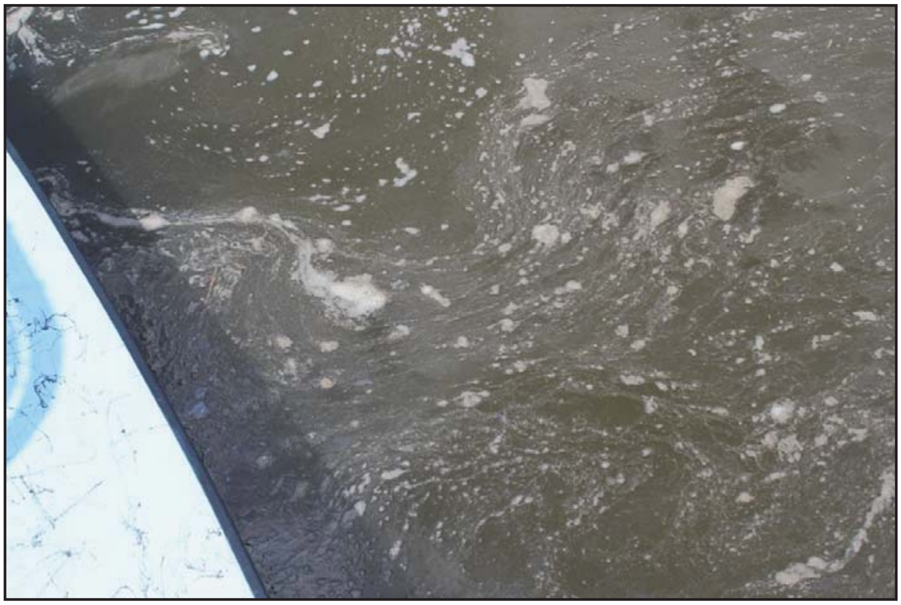

◄ A foamy substance was observed on the surface waters adjacent to emulsified oil suspended below the surface.

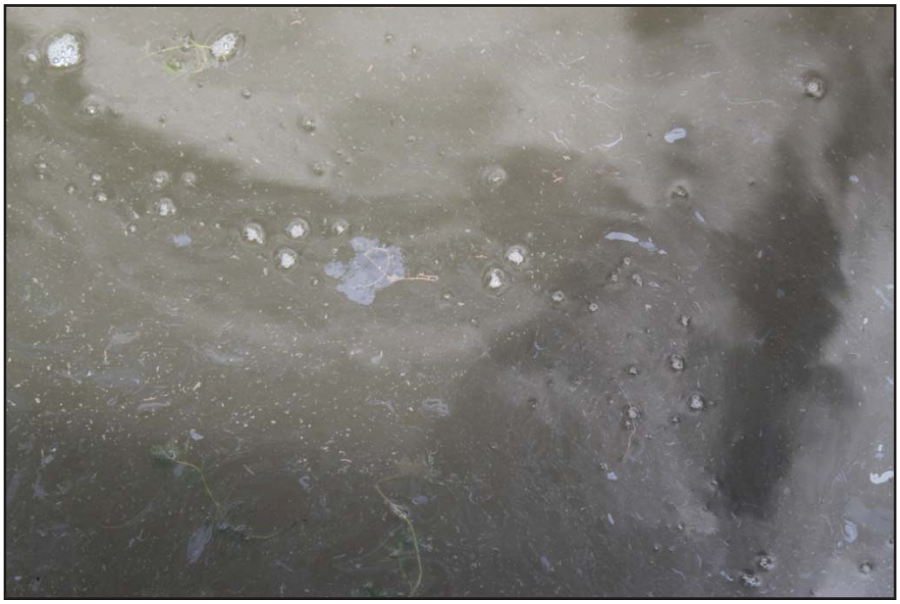

◄ A sheen was present on the surface adjacent to thick brown patches of partially submerged emulsified oil.

FIG. 5 Observation Date – 18 August 2011

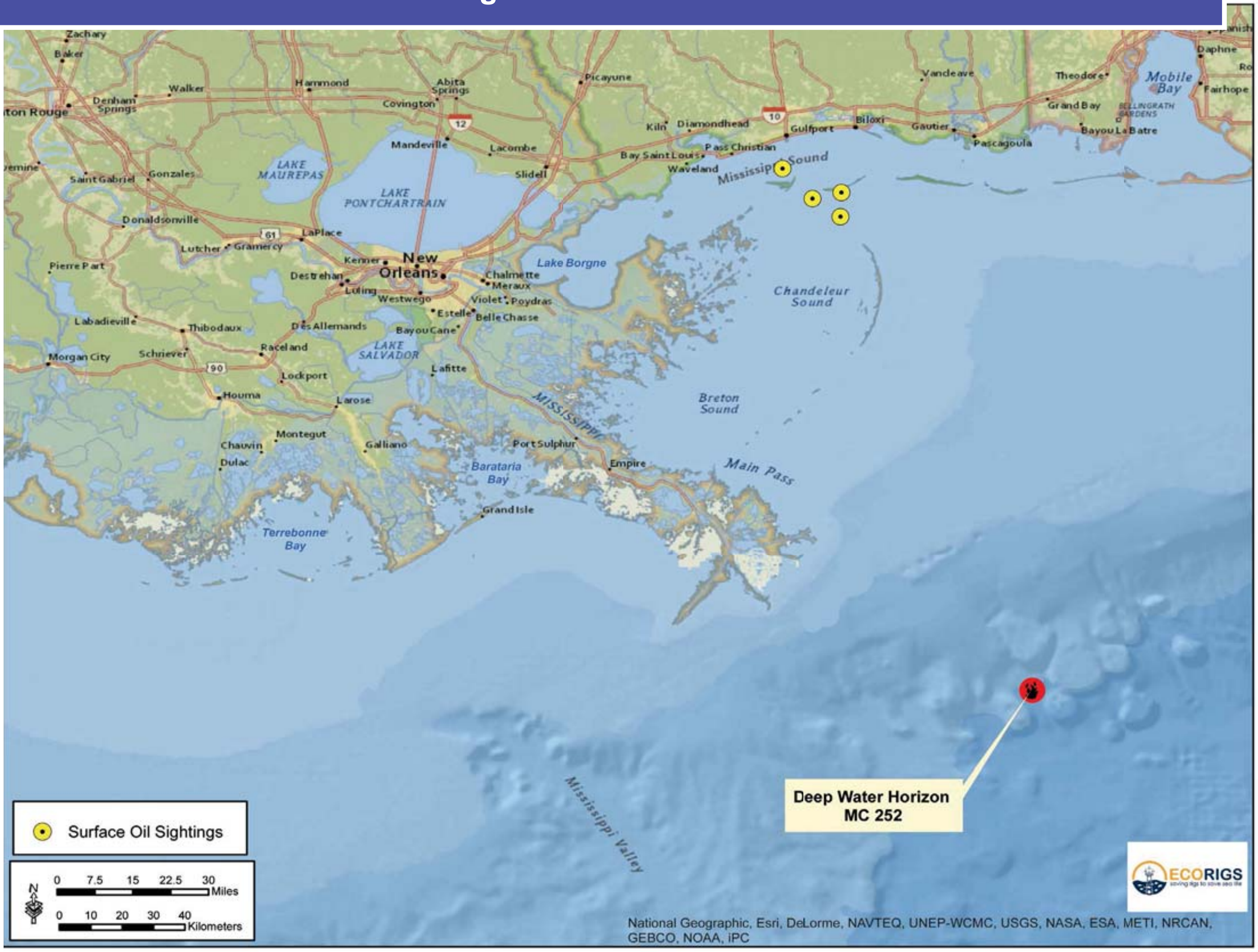

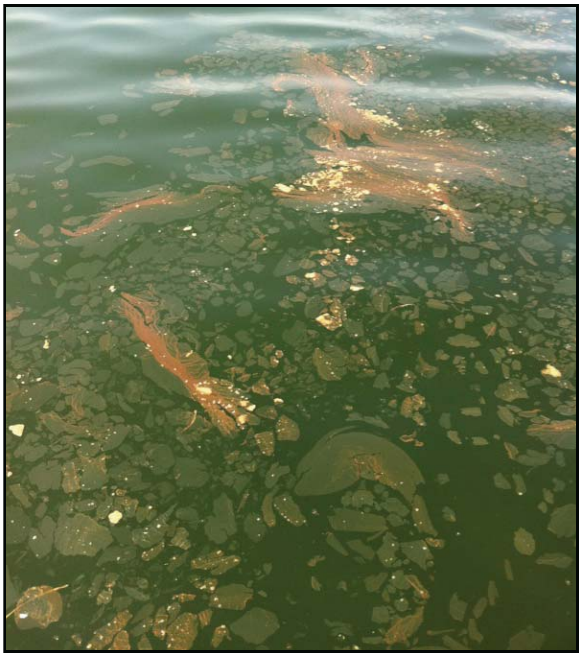

◄ Thick patches of reddish brown crude oil observed on surface waters offshore of Longbeach, MS from 18 August 2011. The patches were also observed near Ship Island, MS.

Observation Date – 18 August 2011 (continued)

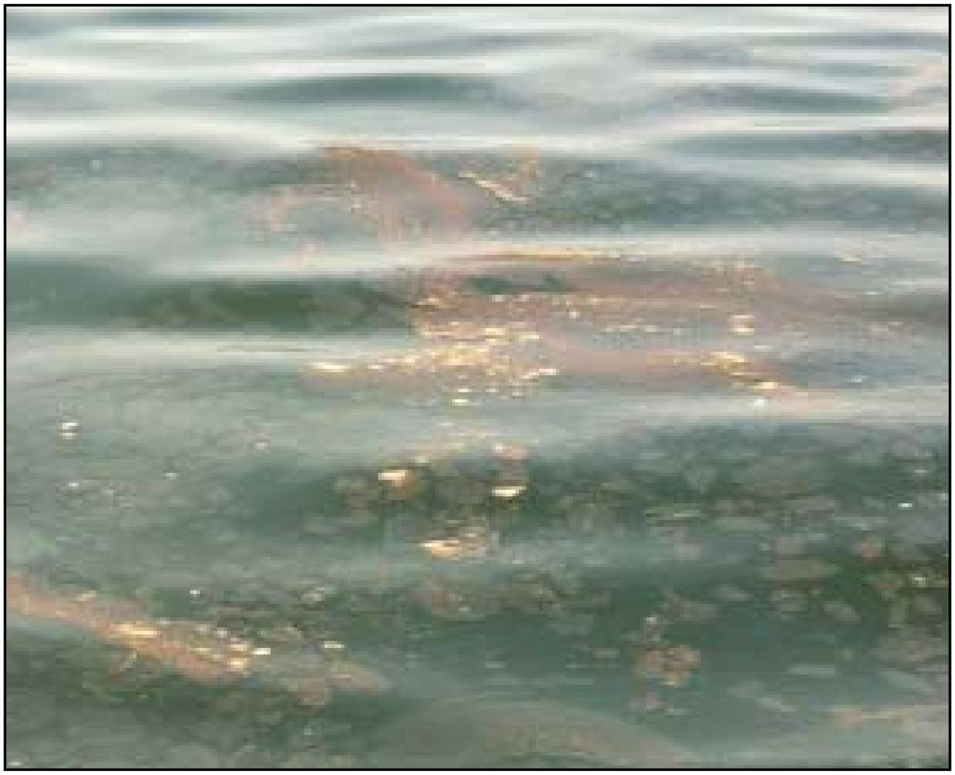

◄ The brown color of the oil is usually associated with emulsification. The seas were calm and the oil is floating and not actively mixing with water.

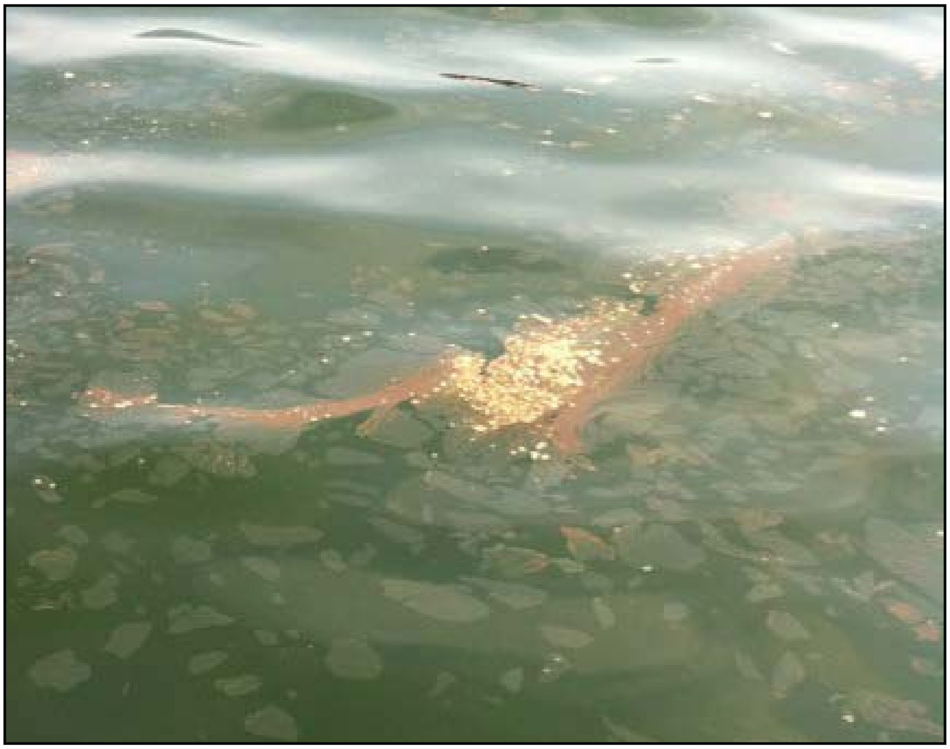

◄ An unknown white substance was present on the oil and it displayed an odd surface expression.

FIG. 6 Observation Date – 14 September 2011

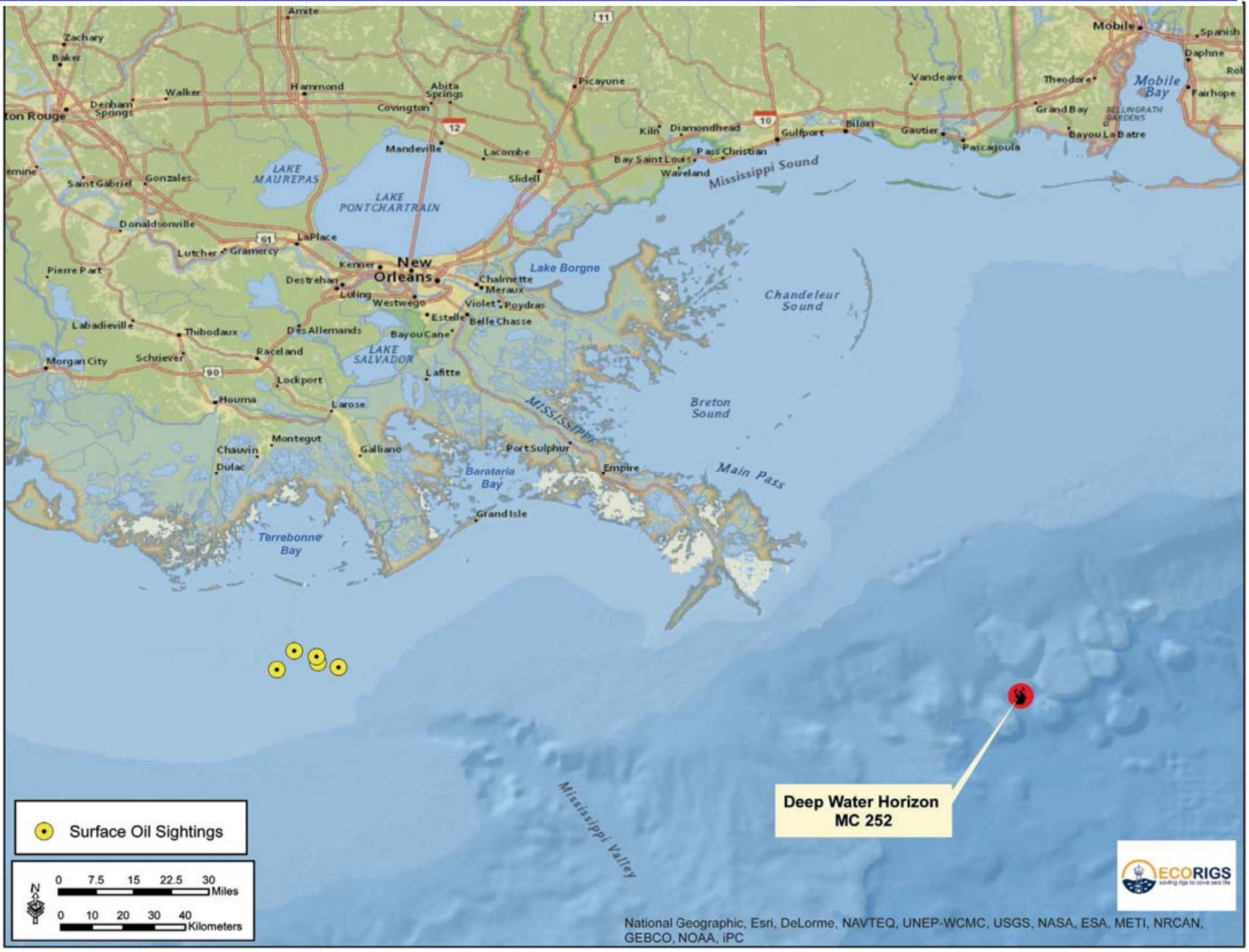

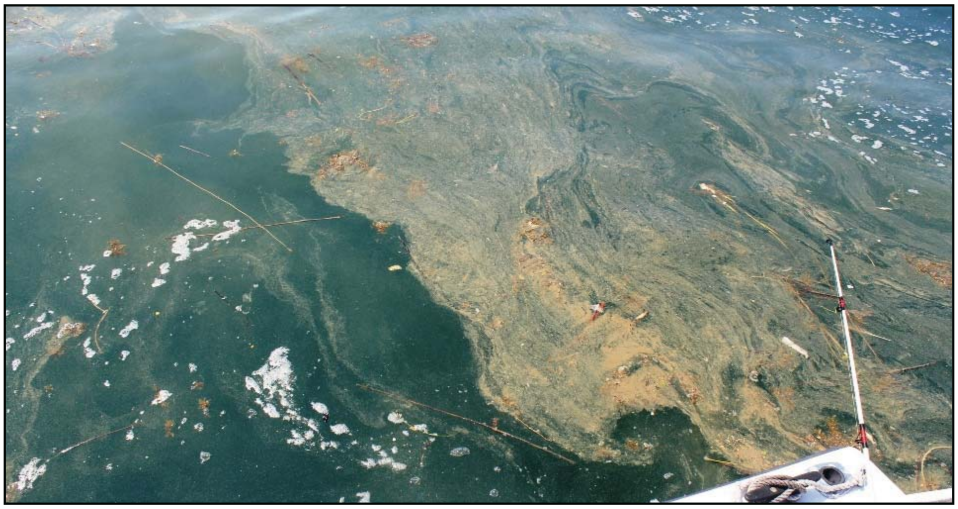

◄ The brown slick appeared 37 km due south of the Houma Navigation Channel. This large slick measured approximately 11 km wide and an unknown length was flowing east to west, parallel to the coast.

Observation Date – 14 September 2011 (continued)

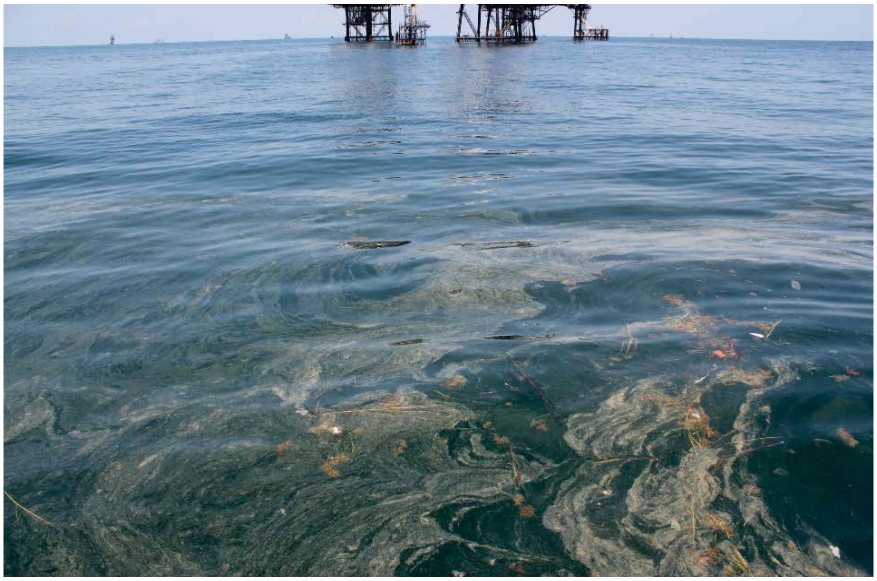

▲ Parts of the of the oil slick were emulsified on the surface and others were partially submerged. The submerged component appeared to be breaking into small droplets.

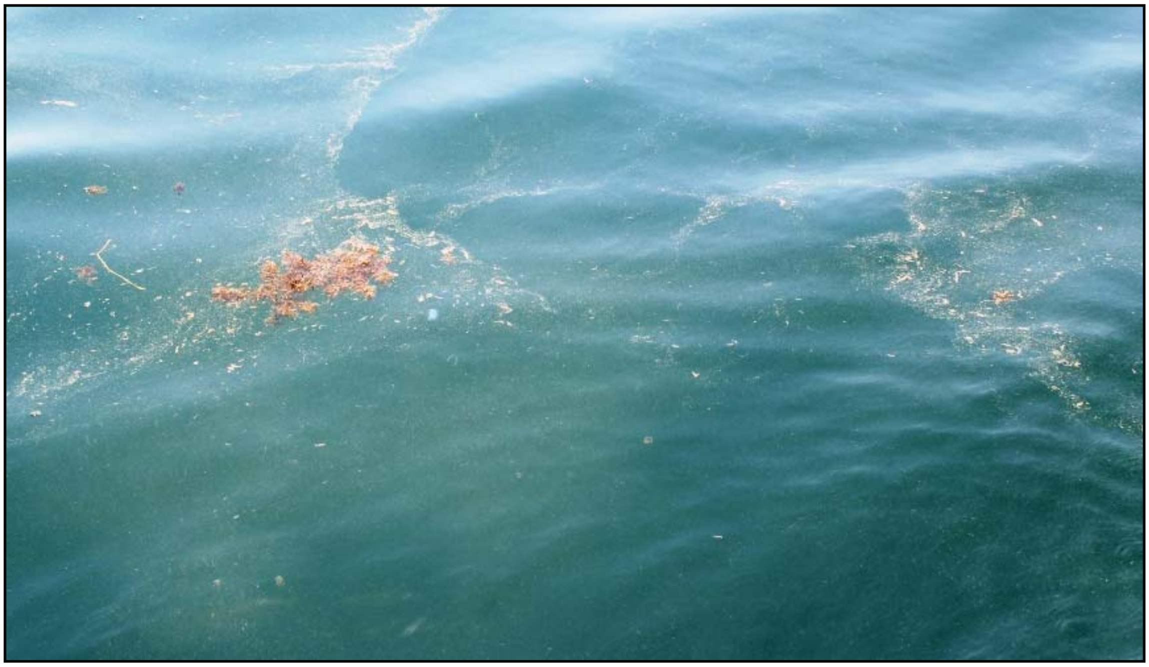

▲ This slick appears to be dispersing from brown emulsified oil into small submerged droplets of oil.

Observation Date – 14 September 2011 (continued)

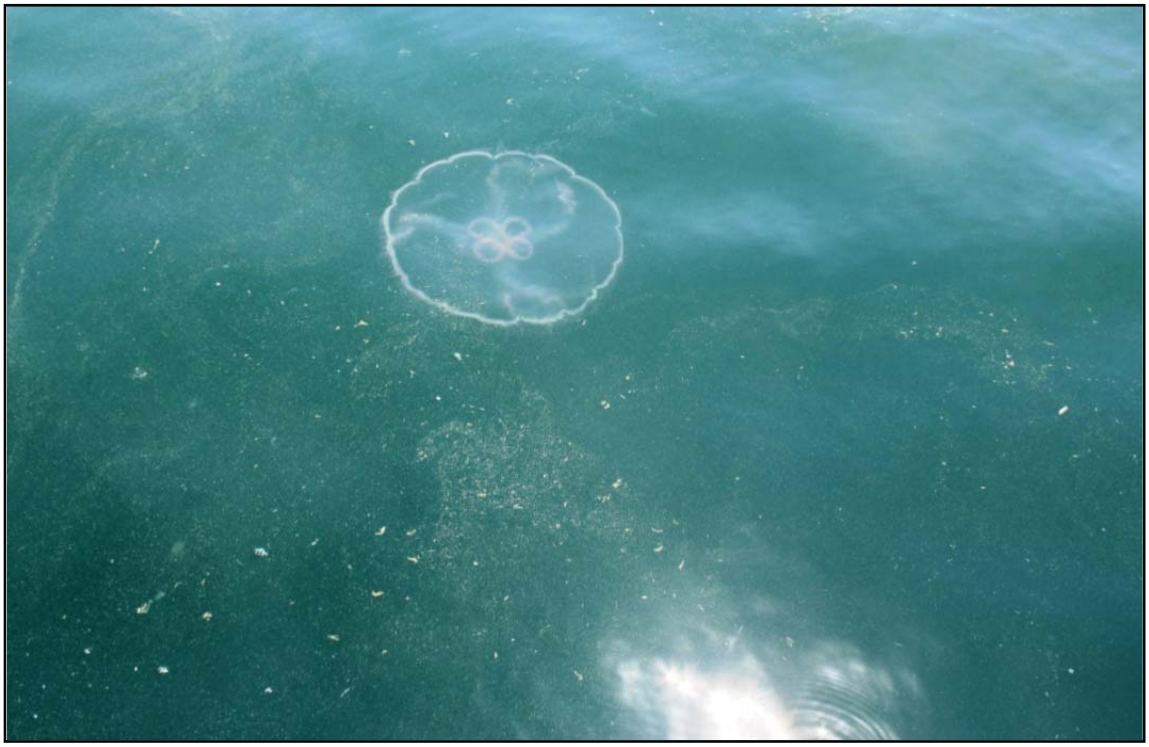

▲ An example of the transition from emulsified oil to dispersed oil. Daling (2011) suggested that MC 252 crude oil transforms from emulsified oil to dispersed oil occurs 3 weeks after release in moderate seas.

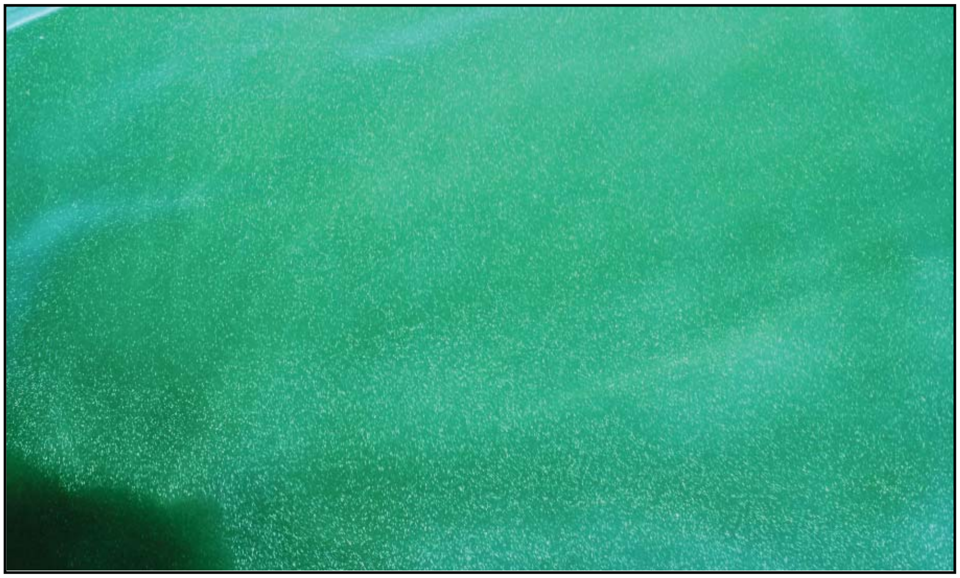

◄ The oil shown in this photograph appears to be dispersed. The floating emulsified oil was present a few km north of the site and now the oil is completely dispersed. The surface is free of oil and the submerged materials in the water column are oil droplets.

FIG. 7 Observation Date – 22 May 2012

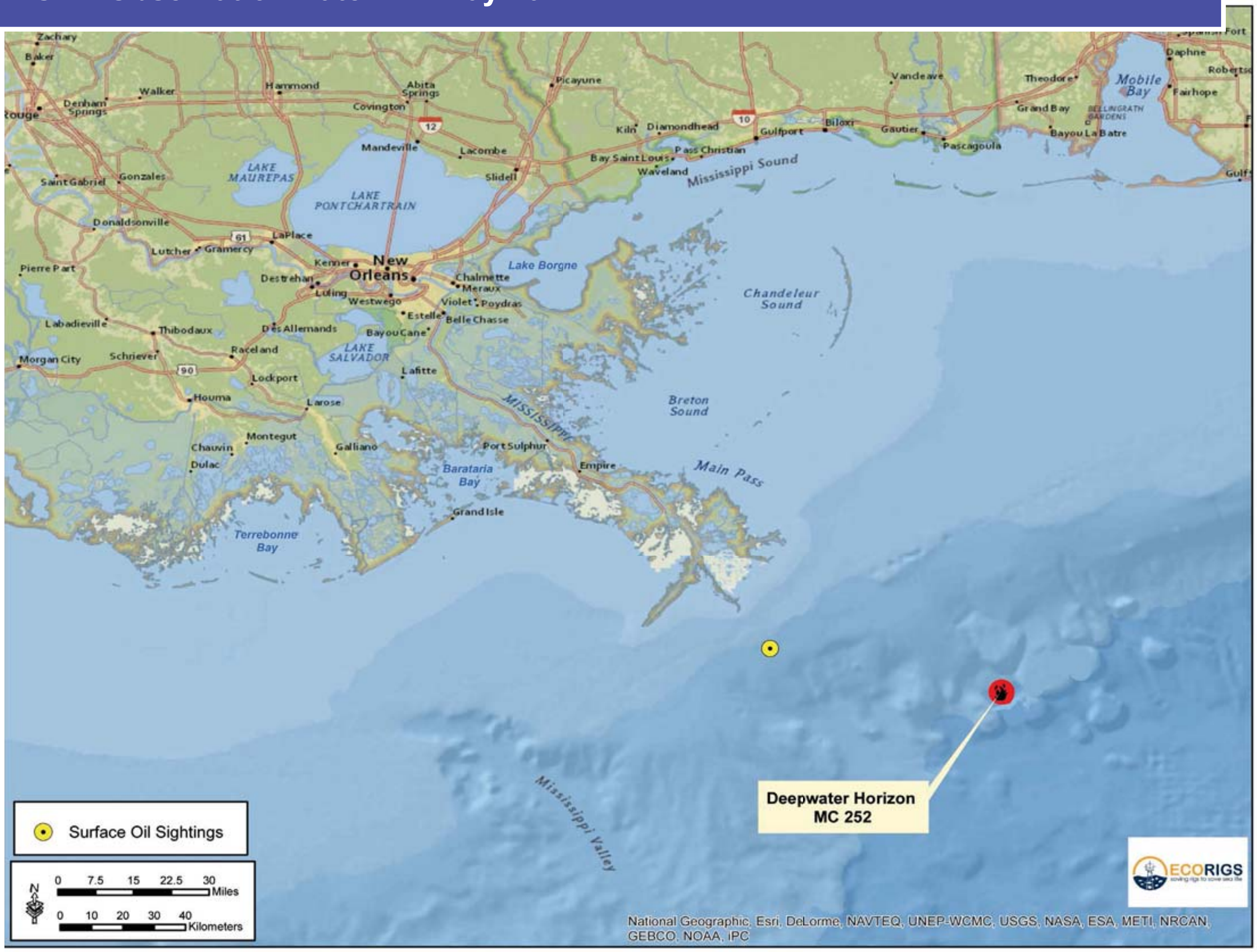

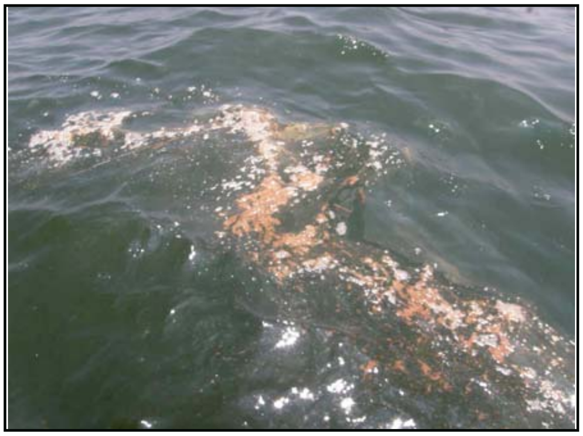

◄ Intermittant patches of reddish brown crude oil was observed offshore of Venice, Louisiana 22 May 2012.

Observation Date – 22-May-2012 (continued)

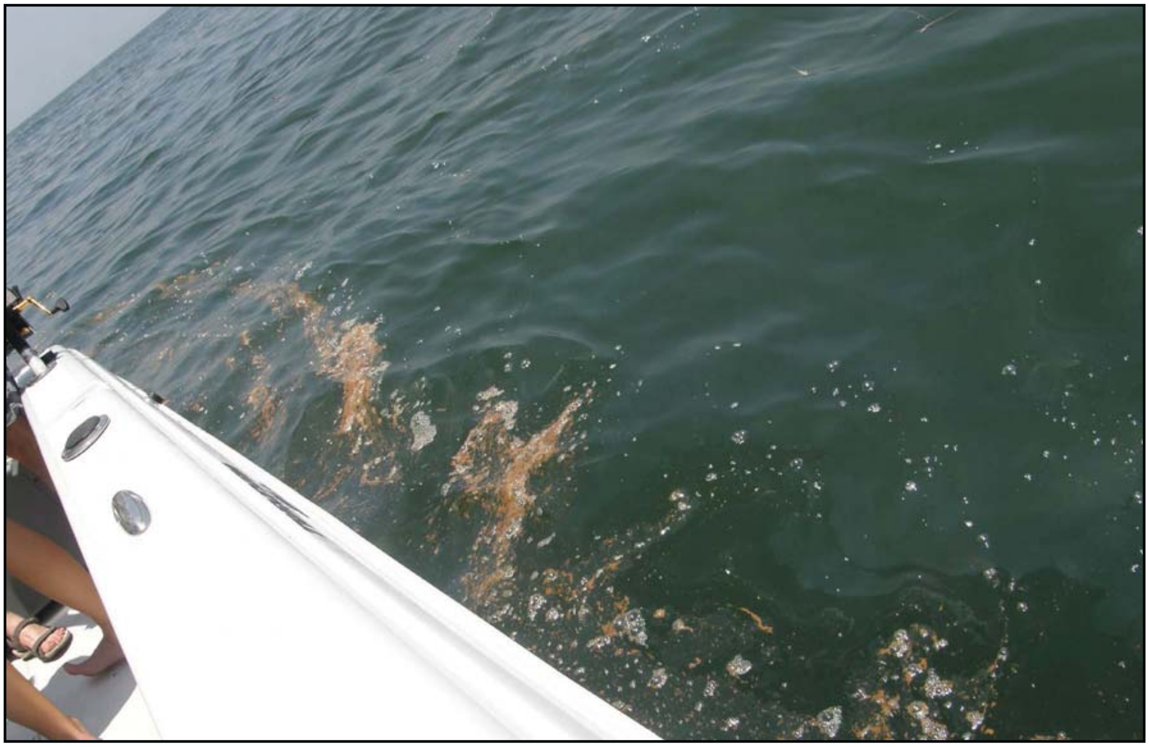

▲ One of several oil slicks passing the vessel on 22 May 2012.

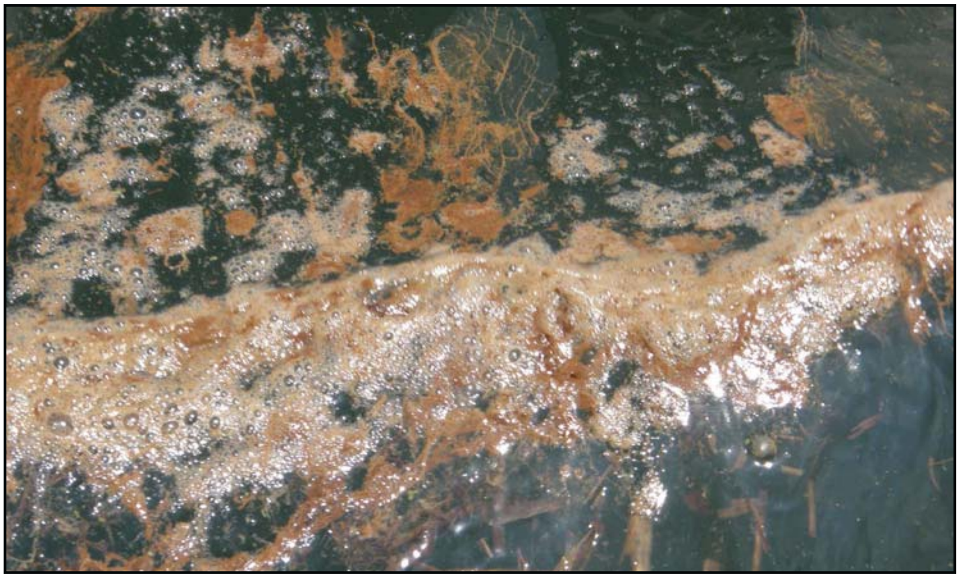

◄ The red-bround emulsified oil is charactoristic of MC-252.

ACKNOWLEDGEMENTS

Figures 1 and 2 were provided with the permission of Comité Européen de Normalisation. Photographs were provided by EcoRigs Non-Profit Organization and location maps by Chris Cothron.

REFERECNES

American Petroleum Institute (1999) Fate of spilled oil in marine waters: An information booklet for decision-makers. API publication no. 4691; Health and Environmental Sciences Department: Washington, D.C; www.ingenieroambiental.com/4001/ACFB2.pdf.

Daling PS (2011) The weathering properties of the Macondo crude at sea in the GoM and the effectiveness of surface and sub-surface dispersant application. Proceedings, CEDRE Information Day. Marine Environmental Technology Dept. SINTEF, Trondheim, Norway.

Belore R, Trudel K, Morrison J, (2011) Weathering, emulsification, and chemical dispersibility of MC-252 crude oil: field and laboratory studies. Proceedings 2011 International Oil Spill Conference. SL Ross Environmental Research.

CEN (Comité Européen de Normalisation) (2011) European Committee for Standardization, Oil spill identification – waterborne petroleum and petroleum products, Part 2: Analytical methodology and interpretation of results based on GC-FID and GC–MS low resolution analyses. FprCEN/TR 155522-2.

Eschenbach TG, Harper VW, Anderson CM, Prentki R (2010) Estimating oil spill occurrence rates: A case study for outer continental shelf areas of Gulf of Mexico. Journal of Environmental Statistics 1, no. 1: 1-19.

International Tanker Owners Pollution Federation (ITOPF) (2002) Fate of marine oil spills. Technical information paper. ITOPF. London, United Kingdom.

MacDonald IR, Guinasso NL, Ackleson SG, Amos, JF, Duckworth R, Sassen R, Brooks JM (1993) Natural oil slicks in the Gulf of Mexico visible from space. J Geophys Res: Oceans (1978–2012), 98(C9), 16351-16364.

MacDonald IR, Reilly JF, Best SE, Venkataramaiah R, Sassen R, Amos J, Guinasso NL (1996) Remote-sensing inventory of active oil seeps and chemosynthetic communities in the Northern Gulf of Mexico. In: Schumacher, D., Abrams, M.A. (Eds.), Hydrocarbon Migration and its Near-Surface Expression. American Association of Petroleum Geologists, pp. 27–37.

Minerals Management Service (2000) Technology assessment of the use of dispersants on spills from drilling and production facilities in the Gulf of Mexico Outer Continental Shelf. MMS Engineering and Research Branch, Herndon, VA. p. 221.

Reddy CM, Arey JS, Seewald JS, Sylva SP, Lemkau KL, Nelson RK, Carmichael CA, McIntyre CP, Fenwick J, Ventura GT, Van Mooy BA, Camilli R (2012) Composition and fate of gas and oil released to the water column during the *Deepwater Horizon* oil spill. Proc. Natl. Acad. Sci. U. S. A. DOI: 10.1073/pnas.1101242108.

Ryerson TB, Aikin KC, Angevine WM et al (2011) Atmospheric emissions from the *Deepwater Horizon* spill constrain air-water partitioning, hydrocarbon fate, and leak rate. Geophys. Res. Lett. 38, L07803.

Sammarco PW, Kolian SR, Warby RA, Bouldin JL, Subra WA, Porter SA (2013) Distribution and concentrations of petroleum hydrocarbons associated with the BP/*Deepwater Horizon* Oil Spill, Gulf of Mexico. Mar. Pollut. Bull. *73*(1), 129-143.
